# A framework for estimating society's economic welfare following the introduction of an animal disease: The case of Johne's disease

**DOI:** 10.1371/journal.pone.0198436

**Published:** 2018-06-06

**Authors:** Alyson S. Barratt, Matthieu H. Arnoult, Bouda Vosough Ahmadi, Karl M. Rich, George J. Gunn, Alistair W. Stott

**Affiliations:** 1 Land Economy, Environment and Society Research Group, Scotland’s Rural College (SRUC), Edinburgh, United Kingdom; 2 School of Agriculture, Policy and Development, University of Reading, Reading, United Kingdom; 3 International Livestock Research Institute (ILRI), East and Southeast Asia Regional Office, Hanoi, Vietnam; 4 Epidemiology Research Unit, Scotland’s Rural College (SRUC), Inverness, United Kingdom; 5 Future Farming Systems Group, Scotland’s Rural College (SRUC), Edinburgh, United Kingdom; University of Illinois College of Veterinary Medicine, UNITED STATES

## Abstract

Animal diseases are global issues affecting the productivity and financial profitability of affected farms. Johne’s disease is distributed on farms worldwide and is an endemic contagious bacterial infection in ruminants caused by *Mycobacterium avium* subspecies *paratuberculosis*. In cattle, the clinical disease manifests itself as chronic enteritis resulting in reduced production, weight loss, and eventually death. Johne’s disease is prevalent in the UK, including Scotland. Direct costs and losses associated with Johne’s disease have been estimated in previous research, confirming an important economic impact of the disease in UK herds. Despite this, the distributional impact of Johne’s disease among milk consumers and producers in Scotland has not been estimated. In this paper, we evaluate the change in society’s economic welfare, namely to dairy producers (i.e. infected and uninfected herds) and milk consumers in Scotland induced by the introduction of Johne’s disease in the national Scottish dairy herd. At the national-level, we conclude that the economic burden falls mainly on producers of infected herds and, to a lesser extent, milk consumers, while producers of uninfected herds benefit from the presence of Johne’s. An infected producer’s loss per cow is approximately two times larger in magnitude than that of an uninfected producer’s gain. Such economic welfare estimates are an important comparison of the relative costs of national herd prevalence and the wider economic welfare implications for both producers and consumers. This is particularly important from a policy, public good, cost sharing, and human health perspective. The economic welfare framework presented in this paper can be applied to other diseases to examine the relative burden of society’s economic welfare of alternative livestock disease scenarios. In addition, the sensitivity analysis evaluates uncertainty in economic welfare given limited data and uncertainty in the national herd prevalence, and other input parameters, associated with Johne’s disease in Scotland. Therefore, until the prevalence of Johne’s is better understood, the full economic cost to Scottish dairy herds remains uncertain but in the meantime the sensitivity analysis evaluates the robustness of economic welfare to such uncertainties.

## Introduction

Animal disease is a global issue affecting the productivity and financial profitability of affected farms. Johne’s disease is distributed on farms worldwide [1] and is an endemic contagious bacterial infection in ruminants caused by *Mycobacterium avium* subspecies *paratuberculosis* (MAP). In cattle, the clinical disease manifests itself as chronic enteritis resulting in reduced production, weight loss, and eventually death [2]. MAP is transmitted from infected cattle to calves within a herd in utero or via manure-contaminated udders, milk, water, or feed [3].

Calves are most vulnerable to becoming infected with MAP and cattle do not typically exhibit clinical signs of the disease until two to five years of age [4]. The long incubation period means the disease is often not detectable in a herd until years after the initial infection [5], the so-called “tip of the iceberg” effect which is common in endemic diseases.

Despite Johne’s global distribution, there are few valid estimates of the prevalence of Johne’s disease in Europe because of problems associated with accurately diagnosing populations [6]. Johne’s is endemic in the UK [7] but there are limited data on its prevalence in the UK. In 2006, Johne’s disease was estimated to have affected 34.7% (95% ci 27.6%-42.5%) of UK herds [8]. While there is much uncertainty surrounding herd-level prevalence, estimates of within herd prevalences are also uncertain. Small scale farm surveys indicate dairy within herd prevalence to be 17.5% ±10% [9,10]. This estimate of prevalence is assumed to be ‘true prevalence’ because it is based on clinical cases diagnosed. However, much uncertainty stems from limited prevalence data and the large number of poorly understood parameters [11].

There is currently no cure for Johne’s disease. Instead, the Scottish Government advises farmers to implement a health and welfare programme in consultation with their veterinarian, to control and prevent infection. The Cattle Health Certification Standards body defines an industry standard screening and control programme which provides a framework for control strategies based on detection by testing and culling of infected animals [12]. However, due to the slow progression of the disease and the lack of accurate diagnostic tools, it can be difficult to diagnose and identify cattle infected with Johne’s [13]. The infection is also of concern for farmers (i.e. producers) because MAP can cause economic losses in affected herds. The production impacts of MAP can result in reduced milk production [14], culling of clinically infected animals [15], increased calving interval (CI), and infertility [16]. Additional treatment and prevention costs originate from the cost of control, monitoring, and diagnosis. Attempts have been made to estimate direct costs and losses associated with Johne’s disease. Bennett and IJpelaar [17] estimated the cost of 34 endemic diseases, of which Johne’s was estimated to be in the range of £0.327-£10 million per year for cattle (i.e. mainly dairy and beef sucker) in Great Britain. Mastitis had the highest cost at around £137–244.7 million per year out of 15 cattle diseases, and all but three of those disease costs exceeded those of Johne’s. Caldow and Gunn [10] estimated direct costs attributable to Johne’s in the UK to be £26 per dairy cow per year. The annual loss to UK beef cattle is estimated to be lower, relative to a dairy cow, at between £10–18 per animal because of a lower prevalence in the beef herd. Stott *et al*. [18] estimated avoidable losses, i.e. the level of expenditure required to minimise the total cost of the disease (output losses and control expenditure) in UK dairy herds to evaluate the financial incentive to control Johne’s. An optimal control strategy focused on culling infected dairy cows reduced the net margin from milk production by £27 per cow annually (i.e. 10%). Hence, the cost estimates for Johne’s are considerably lower than other endemic diseases, suggesting that Johne’s may be of lesser concern financially for producers, providing less incentive to control the disease especially since it is difficult to control. Most cases of Johne’s disease are subclinical, which refers to animals infected with MAP but which do not show clinical signs of the disease [19]. Coupled with this and poor within herd prevalence data make the assessment of economic consequences of MAP difficult. However, better information on the cost of the disease may incentivise improved management of Johne’s disease [18].

The cost estimates provided above demonstrate the economic impact of Johne’s disease on dairy herds. However, the full economic cost of Johne’s disease falls on both producers and milk consumers (because of its effect on milk supply and hence milk prices). Despite this, the relative distributional impact of Johne’s disease among producers and consumers, beyond the farm gate, in Scotland has not been estimated. The economic theory in our model addresses this by assuming that the presence of an animal disease will lead to supply shortage affecting producers and consumers, by increasing the costs of production for farmers and the prices paid for commodities, such as milk, by consumers [20]. In this paper, we evaluate the change in economic welfare to milk consumers and producers (i.e. infected and uninfected herds) at the national-level in Scotland associated with the introduction of Johne’s disease within a single-sector partial equilibrium milk model. While our analysis does not fully capture the broader economic impacts associated with Johne’s, such economic welfare estimates provide initial insights that highlight the relative economic welfare implications in the milk sector amongst consumers and producers.

## Materials and methods

To evaluate the impact of Johne’s disease on economic welfare in Scotland, the total economic welfare (i.e. gains and losses) for three stakeholder groups (i.e. dairy producers with uninfected herds, dairy producers with infected herds, and milk consumers) were simulated following the introduction of Johne’s disease under alternative Johne’s within herd prevalence scenarios (i.e. 7.5%, 17.5% and 27.5%) applied to a single national dairy herd in Scotland. This estimate of prevalence is assumed to be ‘true prevalence’ because it is based on clinical cases diagnosed [9,10]. The economic impact of an outbreak may depend on the size [21] and location [22] of the outbreak. However, the objective of this research was not to consider alterative outbreak sizes, locations or regional effects because only a single national herd is modelled.

Our approach differs from farm-level assessments of the disease [18,23] because it quantifies the wider national-level economic implications of Johne’s. We investigate changes in economic welfare following the introduction of Johne’s disease with no eradication programme. Economic welfare is defined as the total benefit of an action to consumers and producers. A previous study by Weldegebriel *et al*. [24] considered an overnight eradication of bovine viral diarrhoea (BVD) and the resulting gains over a year. However, our approach evaluates the immediate economic welfare impacts associated with the introduction of Johne’s disease modelled on market conditions observed in Scotland because Johne’s is endemic there with little current prospect of eradication (in contrast to BVD). This is relevant because Johne’s disease may affect market conditions, namely production costs, milk prices, and supply. In Scotland, milk quotas were abolished in March 2015 [25], suggesting the milk sector now operates closer to free market conditions [26], which we assume in our model. While retail prices reflect to a certain degree the scarcity of commodities and the consumer response to animal disease outbreaks, nevertheless retailers may absorb some of the cost in response to supply shortages or for other competitive reasons to attract consumers and adjusting margins on other retail offerings [27]. However, this analysis used in this study is based on a theoretical economic framework with assumptions that provide a simplified platform for exploring indirect impacts of endemic disease and the relative importance of their drivers rather than a means to quantify their absolute values. In addition, focusing on the status quo of endemicity in the national-herd means that the costs of an eradication programme are not required. The economic welfare analysis presented here is based on existing market conditions and therefore economic estimates can be directly compared to cost of containment or eradication of a disease such as Johne’s.

### Economic welfare framework

**Fig 1:**
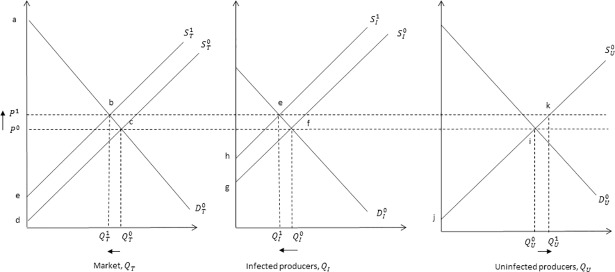
Demand and supply equilibrium associated with reduced milk production following an outbreak of Johne’s. In Fig 1, the intersection of supply curves, $S_{T}^{0}$, $S_{U}^{0}$, and $S_{I}^{0}$, and demand curve, $D_{T}^{0}$, determine the initial equilibrium market price, *P*^*0*^, and quantity supplied by the market, $Q_{T}^{0}$, the infected producers, $Q_{I}^{0}$, and uninfected producers, $Q_{U}^{0}$. A decrease in milk production associated with the introduction of Johne’s shifts the infected producer supply curve backward to $S_{I}^{1}$, the equilibrium quantity supplied by infected producers will decrease, from $Q_{I}^{0}$ to $Q_{I}^{1}$, and the price increases, from *P*^0^ to *P*^1^. Uninfected producers will increase their supply from, $Q_{U}^{0}$ to $Q_{U}^{1}$, in response to a price increase (i.e. a movement along their supply curve). Overall, the total market supply, *Q*_*T* =_ *Q*_*U*_ + *Q*_*I*_, will decrease, from $Q_{T}^{0}$ to $Q_{T}^{1}$, because $\left|Q_{I}^{0}-Q_{I}^{1}|>|Q_{U}^{0}-Q_{U}^{1}\right|$.

The impacts of the disease have wider implications for the economic welfare of milk consumers and producers alike. The presence of Johne’s disease in a herd can result in infected herds not reaching their full production potential relative to an uninfected herd. Since production from an infected herd would be lower than that from an uninfected herd, the quantity of milk supplied to the market would decrease, i.e. a leftward shift in the supply curve of milk. We assume that the incidence of Johne’s reduces the amount of milk produced at any given price, shifting the supply curve to the left and a new equilibrium (Fig 1). In Fig 1, we illustrate this in an equilibrium diagram of milk supply and demand. Consequently, according to economic theory, the market price of milk is likely to increase following a negative supply shock, all else being equal and in the absence of policy distortions or market power that might influence this situation differently. We address these issues later in the Discussion section, focusing our analysis on the “free-market” situation as assumed under neoclassical economic theory.

Economic welfare can be divided into gains for consumers (called consumer surplus) and producers (called producer surplus) [28]. The total economic welfare loss, in this theoretical scenario, is represented by area *bcde* (Fig 1). Consumer surplus is the difference between what milk consumers are willing to pay and the price actually paid for a good or service [29]. Graphically, consumer surplus is the area above the equilibrium price and below the demand curve. Consumers faced with a higher market price (*P*^1^), following a leftward shift in supply associated with a Johne’s outbreak, will experience a loss in economic welfare. As noted in Fig 1, consumer surplus under a disease shock can be denoted by area *P*^1^*ab* in Fig 1 which is smaller than a disease-free scenario (area *P*^0^*ac*). Producer surplus is the difference between the price producers receive and are willing to accept for supply [29]. Graphically, it is the area below the equilibrium price and above the supply curve. As a consequence of reduced milk production associated with Johne’s disease, there will be an overall decrease in producer surplus (from area *P*^0^*cd* to *P*^1^*be*). This loss in producer surplus is made up of a decrease in infected producers surplus (from area *P*^0^*fg* to *P*^1^*eh*) and an increase in uninfected producer surplus (from area *P*^0^*ij* to *P*^1^*kj*).

**Fig 2:**
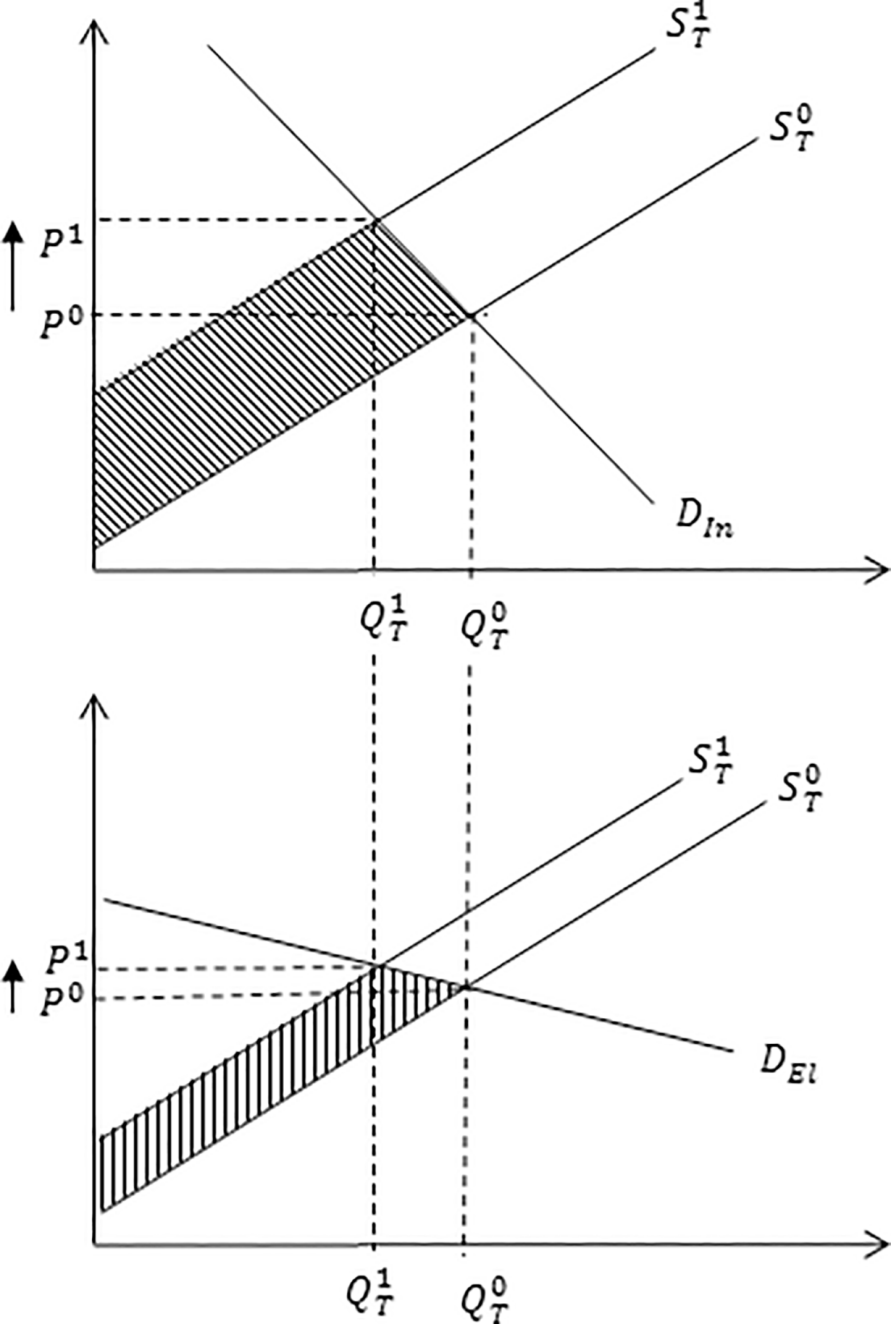
Impact of an inelastic and elastic demand curve on equilibrium market price and quantity. The impact of an inelastic and elastic demand curve on equilibrium market price and quantity associated with a reduction in milk production following an outbreak of Johne’s disease. The inelastic, *D*_*In*_, and elastic, *D*_*El*_, demand curve determine the responsiveness of consumers to new equilibrium market price, *P*^*1*^. A more inelastic demand curve, *D*_*In*_, (i.e. the demand curve is steeper in shape) reflects a larger loss in economic welfare, represented by shaded area, relative to a relatively more elastic demand curve, *D*_*Eln*_, represented by area.

**Fig 3:**
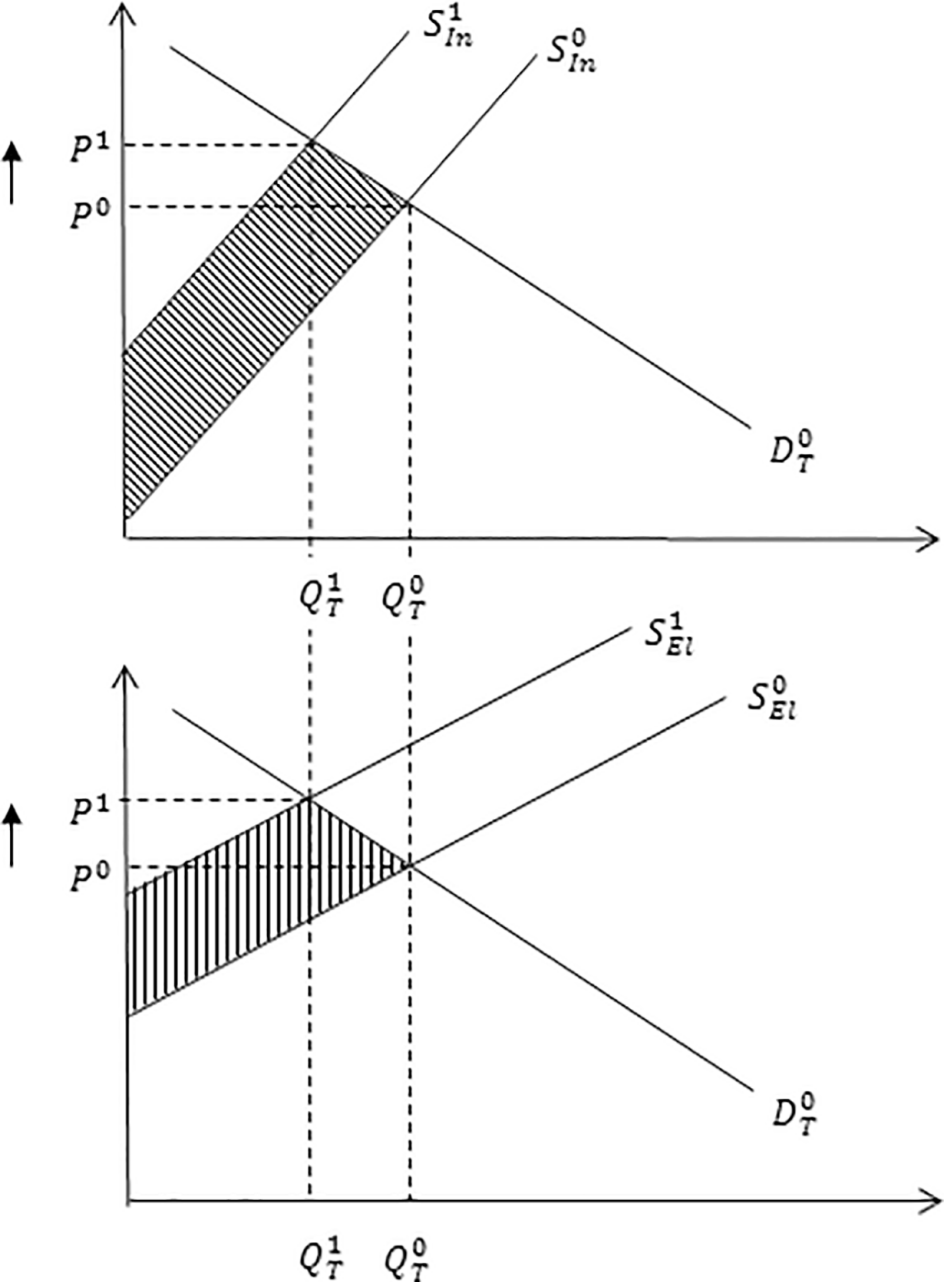
Impact of an inelastic and elastic supply curve on equilibrium market price and quantity. The impact of an inelastic and elastic supply curve on equilibrium market price and quantity associated with a reduction in milk production following an outbreak of Johne’s disease. The inelastic, *S*_*In*_, and elastic, *S*_*El*_, supply curves determine the responsiveness of producers to new equilibrium market price, *P*^1^. A more inelastic supply curve, *S*_*In*_, (i.e. the supply curve is steeper in shape) reflects a larger loss in economic welfare, represented by area, relative to a relatively more elastic supply, *S*_*El*_, represented by area.

Economic welfare estimates are sensitive to model parameters including elasticities of demand (Fig 2) and supply (Fig 3). For instance, for a given leftward shift in supply, an inelastic demand curve has a larger effect on the equilibrium price but a smaller effect on equilibrium quantity, relative to an elastic demand curve. An inelastic demand curve results in a larger loss in consumer surplus, smaller loss in producer surplus and larger loss in total economic welfare, relative to an elastic demand curve (Fig 2). Similar findings hold for when the supply curve becomes more inelastic assuming the supply shock is the same, all else being equal (Fig 3).

### Modelling framework

A Markov-chain (MC) model established the expected annuity from milk production for infected and uninfected herds as a result of Johne’s disease. An economic welfare model, based on the economic framework discussed above, simulated the market-level impacts of Johne’s disease after a year, estimating changes in the price and quantity of milk produced, and the net economic welfare for infected producers, uninfected producers, and milk consumers. Sensitivity analysis evaluated the changes in economic welfare associated with a range of alternative parameter values.

#### Markov-chain model

A MC Microsoft Excel spreadsheet model generated cost estimates for infected and uninfected herds assuming one CI per year. These estimates included costs associated with (i) loss of milk yield; (ii) lost production due to disrupted lactations; (iii) prolonged dry period; (iv) herd age structure; (v) additional culling; and (vi) veterinary care. The procedures for calculating these costs components (i-vi) are described below. The MC model was based on Stott *et al*.’s [18] dynamic programming (DP; [30]) model assuming a 100-cow dairy herd. The DP establishes the sequence of cow replacement decisions in a dairy herd that maximises the expected net present value from milk production. This will depend on the expected net margins from current cows into the future through their consecutive lactations and similarly for their replacements in perpetuity. Given fixed bio-economic assumptions about the parameters which govern these expected net margins (distributions of milk yields by parity, feed costs, culling costs etc.) DP can calculate the long-run (steady state) herd structure and associated financial performance. By adjusting the fixed bio-economic assumptions to represent otherwise identical herds either infected or uninfected with Johne’s disease and comparing them, the unavoidable costs of the disease can be established. This approach recognises the progressive development of the disease through the lifetime of the cow and hence the need in the absence of effective treatments to adjust the replacement decision so that the increasing risks of premature culling and loss due to Johne’s are balanced against the normal costs of culling and the expected changes in yield associated with increasing parity. The MC model described below takes the long-run results from the DP and adds further detail to more fully capture the financial impacts that Johne’s will typically have on an annual basis. These include breaking the annual results from the DP down into quarterly periods so that the disruption caused by unplanned premature culling of Johne’s cases and the slack resources generated (e.g. underutilised buildings and staff) can be properly accounted for. We collectively term these losses ‘opportunity costs’. The MC model also allows exploration of the within lactation yield loss, which was not possible in the annual time steps of the DP. More detailed and realistic culling and veterinary costings were also incorporated. The MC model does not include epidemiological details or capture processes that may be associated with the dynamics of spread of MAP within a herd. Its aim as previously stated is to established the financial impact of on-going Johne’s disease at dairy farm level in line with the wider aims of this paper. A copy of the MC model is available from the corresponding author on request.

#### (i) Loss of milk yield

The in-milk yield loss was based on a MC model developed by Stott *et al*. [18] A binomial distribution modelled the probability of clinical and subclinical cases in a 100-cow herd. The discrete probabilities 0.02 and 0.25 established the probability of clinical and subclinical cases, respectively, in an infected herd [18]. The expected (probability weighted average) herd yield was estimated with these discrete probabilities assuming an average uninfected herd yield of 8,900 litres of milk per cow per lactation. This was estimated by multiplying the average milk yield of 7,893 litres per uninfected cow [31] by the relative yield by lactation number [18] and the distribution of cases by lactation number [32]. The probability of milk yield losses was assumed to be 0.20 and 0.10 of the uninfected instances for clinical and subclinical cases, respectively [18]. Yield loss was converted to a ‘cost’ which deducted 50% of concentrates saved from the proceeds of milk sale, assuming that clinically infected cows continue to eat normally but eat less concentrate because less milk is produced [32].

#### (ii) Opportunity cost within lactation

Involuntary (i.e. unplanned) culling was assumed to disrupt lactation, thereby altering the proportion of the herd in each lactation phase and consequently the yield of the herd overall. This was assumed to be an additional loss of yield experienced by infected cows. Opportunity costs associated with lactation were estimated using the MC model [33]. The CI was split into four equal lactation periods (i.e. early, mid, late, and dry) forming the states of the MC model. Separate MC models were estimated for uninfected and infected herds. In the former, involuntary culling per lactation was assumed to be 0.16 divided equally between the four states [18]. In the infected herd, all clinically infected and 17.6% of sub-clinically infected animals were culled. It was assumed that sub-clinical cases were culled 11% more [34] than the involuntary culling of animals in a disease-free herd (i.e. 16%; [18]). The interval between stages in the MC was 0.25 of a CI to match the states. The involuntary culling rate per state, *i*, is the transition probability to the dry period for all states in the MC. The normal transition probability between one state and the next was given by 1 − *i*. The dry period is an unproductive period associated with involuntary culling representing a delay and disruption associated with idle production factors [32], rather than just the necessary shorter rest period needed by healthy productive animals. This is caused by replacing a cow unexpectedly, as well as the normal dry period after an uninterrupted lactation. The MC model was stationary and the long-run steady state, i.e. the fixed proportion of the herd in the four CI periods of the lactation irrespective of the starting vector, was established for uninfected and infected herds. Using a standard Wood’s curve [35], the loss of milk production due to the effect of Johne’s on the proportion of cows in each part of the curve. We used a 400 day CI and then annualised the financial results for ease of calibration and analysis [36].

Differential CIs for infected and uninfected herds were not considered because CI was only used to annualise results, which otherwise would have been calculated on a CI basis The effects of Johne’s disease on fertility are not clearly ascertained, and the few studies available on the subject are contradictory [37]. Any potential negative effects on fertility were not taken into account to avoid the risk of double counting. As all clinical cows were culled fertility was not a consideration for this cohort.

#### (iii) Opportunity cost of prolonged dry period

Cows in infected herds spent longer in the dry period than cows in uninfected herds based on the differential culling probabilities used in the MC given in the previous section. This lost opportunity cost for production was estimated as the difference between lost milk yield represented by the dry period in infected and uninfected herds. This loss was converted to a lost yield per year and subsequently to a gross margin loss. The gross margin was assumed to be 15.10 pence per litre [38]. The use of a gross margin allowed the lost production to be offset by saved variable costs.

#### (iv) Herd age structure

Premature culling due to Johne’s disease alters the herd age structure, and therefore the production potential due to the effect of parity on yield [32]. DP adjusted voluntary replacements to minimise this effect [18]. However, replacement decisions are not confined to milk yield and expected future Johne’s risk: farmers are unlikely to follow such a policy, even with perfect information. Reliable tests to detect Johne’s disease would encourage farmers to remove infected animals to avoid infecting herd mates, even if the DP decision on an individual cow basis was to keep them.

The long-run steady state herd age structures under fixed voluntary and involuntary culling probabilities for uninfected herds were therefore predicted using the MC model rather than the DP. The aim of this part of the model was to investigate the effect of age structure on the average milk yield of an uninfected herd. The difference between infected and uninfected herd gross margins based on this herd average milk yield difference constituted the lost future income due to Johne’s from herd age structure. The MC model for this exercise used an annual time step of one CI per year and 12 states representing lactation parities 1 to 12 in line with the DP model of Stott et al. (2005). The probability of transitioning between parity *p* to parity *p* + 1 was given by 1 − *r*, where *r* is the replacement probability. For uninfected herds, the replacement probability, *r*, was the probability of involuntary replacement by parity [18,39], with an additional probability of 0.04 representing voluntary replacement. The only exception was in parity 12, in which the probability of replacement was certain. For infected herds the probability of involuntary replacement by parity was adjusted to reflect additional culling resulting from clinical and subclinical cases. To account for this, the conditional probability of a cow being clinical or subclinical for Johne’s within each parity, *p*(*B*|*A*), was estimated. Where *B* is the probability of disease and *A* the probability of being in a given parity. This probability was multiplied by the respective involuntary culling probabilities given above. The estimates of *p*(*B*|*A*) were quantified using the multiplication rule, i.e. *p*(*A*∩*B*)/*p*(*A*) = *p*(*B*|*A*). A distribution of clinical and subclinical cases of Johne’s disease by lactation number [32] together with herd-level probabilities of Johne’s disease cases given above established *p*(*A* ∩ *B*), the probability of a cow being in parity *A* and infected with Johne’s disease. The DP model estimated *p*(*A*), i.e. the distribution of cows in an infected herd by parity under the parameters assumed here including financially optimal voluntary replacement, which established a voluntary replacement rate of 0.04.

Long-run steady state probabilities were estimated for the between herd MCs. Based on the average yields by lactation [18], the average herd yield of an infected herd was estimated to be 0.9975 of an uninfected herd. This difference can be explained by the change in herd age structure associated with premature culling of animals infected with MAP. This parameter was converted to a gross margin (i.e. 0.9975 x 15.10 ppl gross margin as given above) to quantify the financial impact of Johne’s disease on herd age structure.

#### (v) Culling

Culling is comprised of involuntary and voluntary culling. Involuntary culling costs were quantified for the herd age structure of both infected and uninfected herds based on involuntary culling rates by lactation number and reason for culling [39] using the MC model. These estimates were converted into a number of cows per lactation and reason for culling. Cows culled for infertility were sold at their maximum weight-for-age, as predicted by a cow growth model [40]. Other involuntary culling yielded average weights-for-age, as culled cows were not assured of completing lactation and thus gaining weight before sale. For infected herds, extra cows were culled due to additional involuntary culling of clinical and subclinical cases of Johne’s. The latter were culled at average weight-for-age, whereas the former were culled at minimum weight, reflecting the loss of body condition likely in clinically infected animals. This estimated an average weight loss of 79 kg (compared to 100 kg [32]) of a clinical relative to a subclinical cow. A slaughter reduction value of 5% and 30% were applied to the cull value of an uninfected cow to estimate the cull value of a subclinical and clinical cow, respectively [41].

Voluntary culling costs were based on the same age structure as for involuntary culling. Additional costs due to MAP were due solely to differences in age structure between the uninfected and infected herds. This was because voluntary culling rates and values of culled animals were assumed to be identical in uninfected and infected herds.

#### (vi) Veterinary costs

Each clinical case incurred a veterinary cost of £100 per cow, this estimate included veterinary call-out charges, examinations, palliative treatment and blood testing [18].

#### Economic welfare model

An economic welfare model in R [42] simulated the changes in economic welfare associated with the introduction of Johne’s disease at the national-level in Scotland after a year. The economic surplus for producers of infected herds, producers of uninfected herds, and milk consumers in Scotland was estimated using the estimate of total costs excluding yield loss (Table 1), economic model parameters (Table 2), and the prices and quantities described below.

**Table 1:** Costs associated with the presence of Johne’s disease.

Source of cost	£ per infected cow per year	€ per infected cow per year^a^
Milk yield loss	60.57	70.73
Opportunity cost within lactation	1.36	1.59
Opportunity cost of a prolonged dry period	2.38	2.78
Herd age structure	2.26	2.64
Culling		
Involuntary culling	-6.87	-8.03
Voluntary culling	51.19	59.78
Veterinary cost	2.00	2.3354
Total cost	112.89	131.82
Total cost excluding milk yield loss	52.31	61.09

^a^ 1.37766 average British pound to euro currency exchange rate over 2015 [43]

**Table 2:** Economic welfare model input parameters.

Parameter (units)	Value	Source/Estimation
Price elasticity of supply of milk, *ε*	1.759	[44]
Price elasticity of demand of milk, *η*	-0.2198	[45]
Households in Scotland	2,419,921	[46]
Scottish population	5,347,600	[47]
Scottish dairy herd size (number of cows)	175,734	[48]
National herd prevalence of Johne's disease (%)	17.50	[10]
Size of infected herd (number of cows)	30,753	= 0.175* 175,734
Size of uninfected herd (number of cows)	144,981	= (1–0.175)*175,734
Milk yield of an uninfected cow (litres per cow per year)	7,893	[31]
Difference in milk yield of an infected cow relative to an uninfected cow (litres per cow per year)	-192	[18]
Milk yield of an infected cow (litres per cow per year)	7,701	= 7,893–192
Initial total milk yield of all uninfected cows (litres)	1,144,335,033	= 144,981*7893
Initial total milk yield of all infected cows (litres)	236.828,853	= 30,753*7701
Initial price of milk (pence per litre)	26.50	[38]

#### Price and quantity of milk

Market clearing was assumed such that the quantity supplied equalled quantity demanded of milk. The supply and demand functions were assumed to be linear around the initial equilibrium, where price and quantity changes are assumed to be small [49]. A reduction in the supply of milk (i.e. a parallel leftward shift in supply) (Fig 1) following the introduction of Johne’s disease, but no change in demand was assumed. In the base year, *t*_0_, the size of the Scottish dairy herd was defined as 175,734 animals [48]. In the following year, *t*_1_, Johne’s was introduced assuming two national herds, an infected and uninfected herd, defined by a national herd prevalence of 17.5%.

The initial price of milk in period *t*_0_, *P*, was based on a farm gate price of milk of 26.5 pence per litre[38]. The initial quantity of milk produced (1,381,156,725 litres) was estimated in the economic welfare model based on an aggregation of the milk produced by all infected and uninfected cows. The initial quantity of milk produced by infected cows at the national-level was estimated by expressing the number of infected cows in terms of litres of milk based on yield generated from the aforementioned MC model. Milk produced by the infected herd at the national-level was aggregated assuming that any yield loss induced by Johne’s was uniform across the national herd in Scotland. This implicit assumption was based on a survey of veterinary experts in Scotland [50] and communication with experts in Europe [51]. A minority of experts in this Scottish survey ranked herd size as an important risk factor. This opinion is reiterated by stakeholders in the USA, where herd size is identified as an epidemiological risk factor which significantly impacts the spread of MAP [52]. However, there is no evidence to support the impact of herd size in Scotland and so herd size was assumed to have no effect.

Changes in the price and quantity of milk, induced by the introduction of Johne’s disease, were estimated over one year because MAP can survive in the environment for up to a year [53]. Furthermore, the analysis is focussed on the changes in short-term, i.e. a year, because the demographic structure of an infected and uninfected herd is likely to change beyond that time horizon. Havrila and Arch’s [54] marginal analysis method was adopted to estimate changes in price and quantity over a one-year period for three stakeholder groups; producers with infected herds, producers with uninfected herds, and milk consumers. The impact of the introduction of Johne’s on equilibrium price and quantity was estimated by simultaneously solving the following system of Eq (1–4) for two periods (before Johne’s and after) [54]:

$$\left(\frac{1}{\varepsilon }\right)\left(\frac{P}{Q_{I}}\right){dQ}_{I}-dP={MC}_{I}da_{I}$$ (1)

$$\left(\frac{1}{\varepsilon }\right)\left(\frac{P}{Q_{U}}\right){dQ}_{U}-dP=0$$ (2)

$$\left(\frac{1}{\eta }\right)\left(\frac{P_{t}}{Q_{C}}\right){dQ}_{C}-dP=0$$ (3)

$${dQ}_{I}+{dQ}_{U}-{dQ}_{C}=0$$ (4)

In Eqs (1)–(4), the price elasticity of demand [55] for milk, *η*, measures the extent to which changes in the price of milk are associated with changes in the quantity of milk demanded. The price elasticity of supply [55] for milk, *ε*, measures the extent to which changes in the price of milk are associated with changes in the quantity of milk supplied. The quantities of milk produced by infected producers, uninfected producers, and consumed by consumers in period *t*_0_ are denoted by *Q*_*I*_, *Q*_*U*_, and *Q*_*C*_ respectively. The change in the quantity of milk produced by infected, *dQ*_*I*_, uninfected, *dQ*_*U*_, and consumed by consumers, *dQ*_*C*_, in period *t*_1_, equates to zero. Market clearing is assumed and it is also assumed that producers maximise profit by adjusting output until marginal cost of milk of infected producers, *MC*_*I*_, equals the price of milk, *P*. The shifter, *a*_*I*_, determines the impact of the proposed policy of the marginal cost function. In this case study, it is assumed that the introduction of Johne’s will affect both milk yield and price. Given this assumption, the change in marginal cost is:

$${MC}_{I}{da}_{I}=[P({dY}_{I}/Y_{I})-dC/Y_{I}]/[1-dY/Y_{I}]$$ (5)

where *dY* is the difference in milk yield of an infected animal, *Y*_*I*_, relative to the yield of an uninfected animal *Y*_*U*_, and *dC* denotes the change in cost of an infected animal relative to an uninfected cow.

#### Economic welfare

The impact of Johne’s on the economic welfare for each of the three groups (i.e. infected producers, uninfected producers and milk consumers) was based on a methodology initially presented by Lichtenberg *et al*.[49], and later refined by Andersson *et al*. [56], Ebel *et al*. [57], and Forsythe and Corso [58]. Following changes in market price and quantity, changes in economic welfare were quantified where changes in surplus accruing to infected dairy producers (Δ*PS*_*I*_); uninfected dairy producers (Δ*PS*_*U*_); and milk consumers (Δ*CS*_*C*_) in period *t* were defined as:

$${\Delta PS}_{I}=(P+dP)(Q_{I}+{dQ}_{I})-PQ_{I}-\frac{dC}{Y_{I}}(Q_{I}+{dQ}_{I})-P{dQ}_{I}-{\frac{P({dQ}_{I})}{2\varepsilon Q_{I}}}^{2}$$ (6)

$${\Delta PS}_{U}=dP\left(\frac{2Q_{Ut}+{dQ}_{U}}{2}\right)$$ (7)

$${\Delta CS}_{C}=-dP\left(\frac{2Q_{C}-{dQ}_{C}}{2}\right)$$ (8)

where the change in marginal cost per yield, *dC*/*Y*_*I*_, denotes the change in the production cost of milk, *dC*, per unit of output from an infected herd. The terms *PdQ*_*I*_ and *P*(*dQ*_*I*_)^2^/2*εQ*_*I*_ denote the cost savings for producers of infected herds, due to the reduction in the level of output, *dQ*_*I*_, arising from Johne’s. The total economic welfare loss for Scotland is the sum of economic welfare changes of infected producers, uninfected producers, and milk consumers.

### Sensitivity analysis

The sensitivity to changes in economic welfare for each of the three stakeholder groups was evaluated using a range of parameter values obtained from the literature. Elasticities of supply were assumed to range from 1.5 to 2.0 [59] while the elasticity of demand ranged from -0.45 to 0.00 [60]. The national herd prevalence of Johne’s disease was 17.5% ±10%, i.e. three alternative national herd prevalence scenarios (7.5%, 17.5% and 27.5%) were evaluated. The national herd herd-level prevalence parameter was extended to range from 0–100% to account for all scenarios [11].

## Results

Net economic surplus by stakeholder group (i.e. infected producers, uninfected producers and milk consumers) in Scotland is presented in Table 3. Economic welfare analysis indicates an overall loss for Scotland as a consequence of the introduction of Johne’s in the national herd. On aggregate milk consumers experience the largest economic loss associated with the introduction of Johne’s, but infected producers also incur economic losses at the national-level. Only uninfected producers gain from the introduction of Johne’s disease, but these gains do not offset the economic welfare losses incurred by infected producers or consumers.

**Table 3:** Net economic surplus for stakeholder groups (i.e. infected producers, uninfected producers, consumers) and Scotland in a year following the introduction of Johne’s under alternative national herd prevalence scenarios (i.e. 7.5%, 17.5% and 27.5%) in million pounds [million euros^a^].

National herd prevalence (%)	Infected producers	Uninfected producers	Consumers	Scotland
	£M [€M]	£M [€M]	£M [€M]	£M [€M]
7.5	-0.60 [-0.83]	1.10 [1.51]	-1.18 [-1.63]	-0.69 [-0.95]
17.5	-1.15 [-1.58]	2.30 [3.16]	-2.76 [-3.80]	-1.61 [-2.22]
27.5	-1.39 [-1.92]	3.19 [4.40]	-4.33 [-5.97]	-2.53 [-3.49]

^a^ 1.37766 average British pound to euro currency exchange rate over 2015 [43]

Net economic surplus expressed per cow and per household are reported in Table 4. Gains and losses incurred by producers of uninfected and infected herds, respectively, indicate the distributional burden of Johne’s borne by the industry following the introduction of Johne’s. Therefore, the economic welfare figures (Table 4) provide a useful estimate as to the relative cost of Johne’s ‘per cow’ on which to estimate gains or losses in a herd of any given size. The overall “all producer” figure suggests an average gain associated with Johne’s disease per cow regardless of a producer knowing the Johne’s disease status of their herd. An infected producer’s loss per cow is approximately two times larger in magnitude than that of an infected producer’s gain.

**Table 4:** Net economic surplus for stakeholder groups (infected producers, uninfected producers, consumers) and Scotland in a year following the introduction of Johne’s, per cow and household under alternative Johne’s disease national herd prevalence scenarios (i.e. 7.5%, 17.5% and 27.5%) in pounds [euros^a^].

National herd prevalence (%)	All producers	Infected producers	Uninfected producers	Consumers
	£ [€] per cow	£ [€] per infected cow	£ [€] per uninfected cow	£ [€] per household
7.5	2.80 [43.86]	-19.65 [-27.07]	7.57 [10.42]	-0.49 [-0.67]
17.5	6.53 [8.99]	-37.38 [-51.49]	15.84 [21.82]	-1.14 [-1.57]
27.5	10.25 [14.10]	-45.25 [-62.32]	22.01 [30.32]	-1.79 [-2.47]

^a^ 1.37766 average British pound to euro currency exchange rate over 2015 [43]

**Fig 4:**
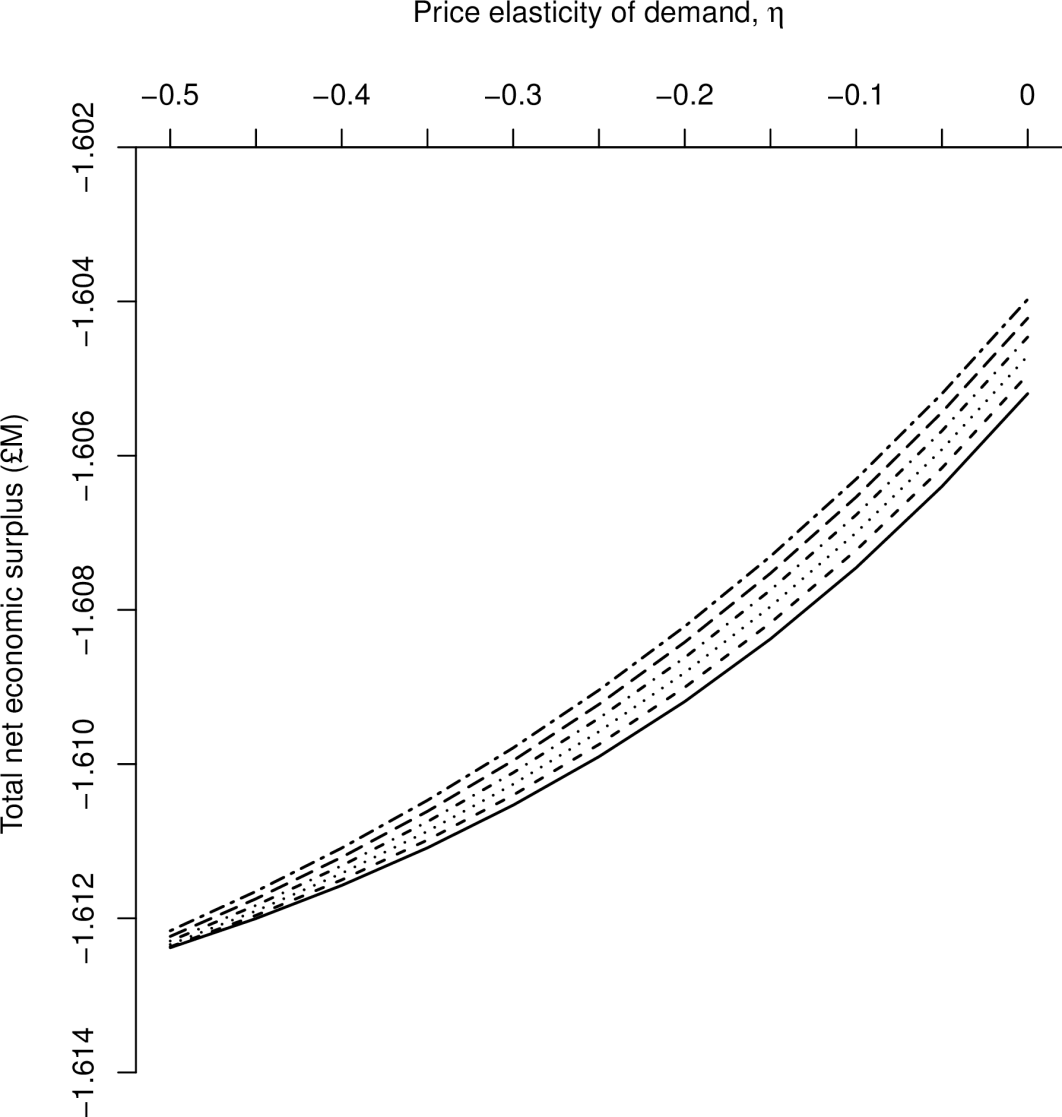
Sensitivity of net economic surplus for Scotland to elasticity of demand and supply. The sensitivity of aggregated net economic surplus (million £) for Scotland following an outbreak of Johne’s with respect to variation in the elasticity of demand, *η*, (-0.50 to 0.00), and elasticity of supply, *ε*, (1.5; 1.6; 1.7; 1.8; 1.9; 2.0).

The sensitivity of Scotland’s net economic surplus to changes in demand and supply elasticities at the national-level is presented in Fig 4. For a given elasticity of supply, the Scotland-level net economic surplus loss decreases as demand becomes more inelastic (*i*.*e*., a rightward shift along the horizontal axis). In addition, for a given elasticity of demand, Scottish-level net economic surplus increases as supply becomes more elastic because the quantity supplied changes more than proportionately to a given percentage change in price.

**Fig 5:**
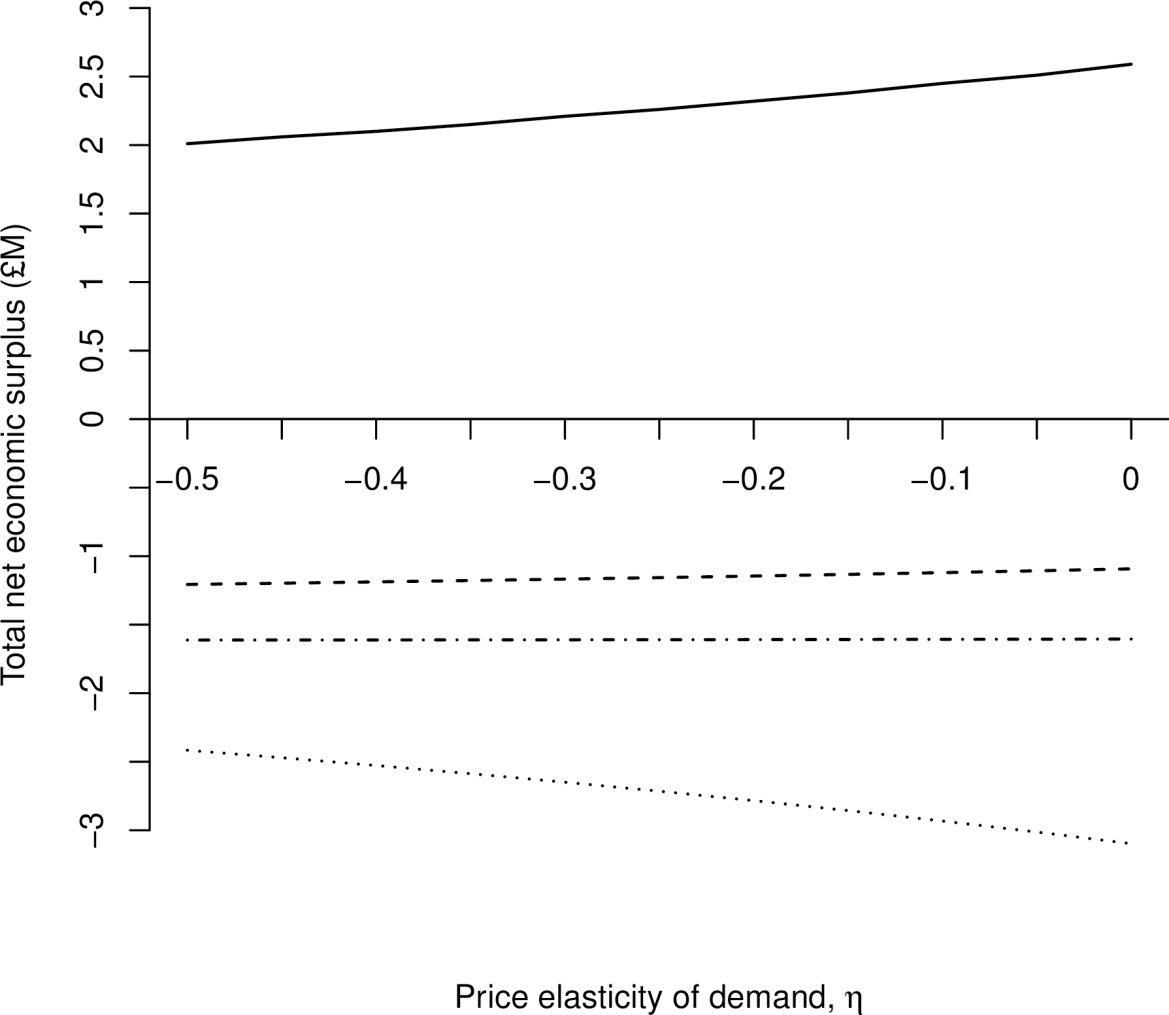
Sensitivity of net economic surplus to the elasticity of demand by stakeholder group. The sensitivity of net economic surplus (million £) to elasticity of demand by stakeholder group (i.e. uninfected producers; infected producers; consumers; Scotland) following an outbreak of Johne’s with respect to a constant elasticity of supply, *ε*, (1.759), and a variation in the elasticity of demand, *η*, (i.e. -0.45 to 0.00).

Net economic surplus disaggregated by the three stakeholder groups with respect to variation in the elasticity of demand (-0.5 to 0.00), assuming a constant elasticity of supply (1.759) [44] and a constant national herd prevalence (17.5%) is presented in Fig 5. Changes in economic surplus in response to changes in elasticity of demand are relatively modest and quasi linear. As demand becomes more elastic, (i.e., leftward shift along the horizontal axis), the surplus gain of producers with uninfected herds decreases, almost mirroring the rate at which consumer surplus losses decreased. Infected producer surplus remains relatively constant as the elasticity of demand becomes more elastic, decreasing at the same rate as the surplus loss at the national-level for Scotland. As the price elasticity of demand for milk becomes more elastic, consumers will react more responsively to a price increase by buying proportionately less milk, thus decreasing the volumes produced and traded.

Net economic surplus disaggregated by the three stakeholder groups with respect to variation in the elasticity of supply (1.5 to 2.0), assuming a constant elasticity of demand (-0.2198) and a constant national herd prevalence (17.5%) is quasi-linear and exhibits no variation.

**Fig 6:**
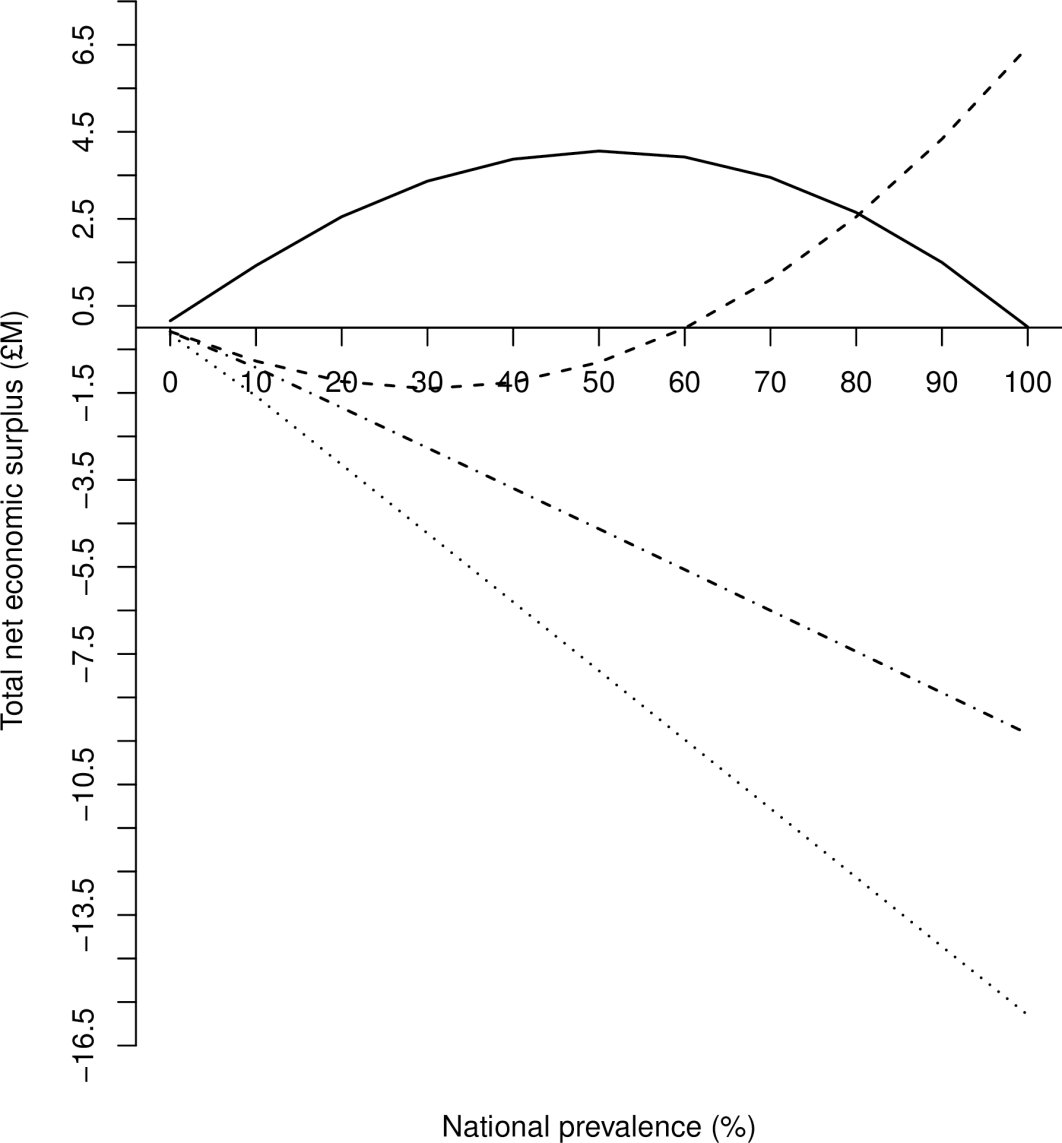
Sensitivity of net economic surplus to national herd prevalence by stakeholder group. The sensitivity of net economic surplus (million £) to national herd prevalence by stakeholder group (i.e. uninfected producers; infected producers; consumers; Scotland) following an outbreak of Johne’s with respect to a constant elasticity of demand, *η*, (-0.2198), and elasticity of supply, *ε*, (1.759).

In Fig 6, we highlight the sensitivity of net economic surplus among the three stakeholder groups to alternative levels of national herd prevalence of Johne’s (0–100%) in Scotland, assuming constant elasticities of demand (-0.2198) and supply (1.759). As the national herd prevalence increases, alternative economic surplus trajectories emerged for each stakeholder group, illustrating the degree to which the distribution of surplus varies with national herd prevalence amongst the three stakeholder groups.

Uninfected producer surplus increases until 50% of the national herd are infected. Beyond this point, infected producer surplus gains diminish to zero as national herd prevalence approaches 100%. Uninfected producers benefit from a higher price due to restricted output, while not being burdened with extra production costs associated with Johne’s. While this remains true with any given level of national herd prevalence in the national herd, as the number of uninfected producers decreases, MAP spreads to more animals, suggesting that the surplus aggregated over all uninfected producers eventually reaches zero when there few remaining uninfected animals in the country.

Producers of infected herds experience a maximum loss in economic welfare at a national herd prevalence of 30%. As the number of infected animals increase, with each producer restricting output and facing increased production costs, this will offset the benefit of a higher market price. Infected producer surplus increases, beyond the point where more than 60% of the national herd is infected, because infected producers contribute to a larger share of national herd and total output and so production does not expand so much.

The increase in milk consumer surplus losses linearly declines with increasing national herd prevalence. Consumers lose out from the occurrence of Johne’s, as a reduced supply and higher production costs associated with the disease drive prices upwards (Fig 1). At the national-level, a linear decrease in economic welfare loss is observed, the majority of which is attributable to the economic loss associated with producers of infected herds.

## Discussion

Overall, the net economic welfare at the national-level associated with a constant national herd prevalence of Johne’s disease was negative. On aggregate, milk consumers bear the greatest loss when faced with a reduced quantity and higher price of milk. In practice, the extent to which price adjustments are passed onto the consumer will vary depending on the magnitude of domestic supply reduction and supply destined for export which could cover shortfall in the domestic supply [27]. Moreover, the degree of market power in retail could also play an important factor. Individual retailers may choose to absorb a portion of the price increase associated with Johne’s, while offsetting those losses by raising prices on other retail offerings. In Scotland, milk is often marketed as a loss leader, being sold below marginal cost to attract consumers into the store. In the past, retailers have been investigated and fined for price-fixing [61]. Hence the price of milk does not necessarily reflect the true cost of production but is likely to be absorbed somewhere along the supply chain. Our economic welfare model estimated consumer milk prices increased by only 0.752% in response to Johne’s disease. Assuming that milk retails at 26 pence per litre [62], a 0.752% increase in the price of milk translates to only a 0.192 pence increase per litre. In Scotland, 92% of Scotland’s dairy products are sold domestically within the UK [63]. Furthermore, Johne’s disease is not a notifiable disease [12], which means milk trade is not restricted and unlikely to be affected by the disease Hence, spillover effects on welfare and trade are likely to minimal and restricted to the domestic market. The wider distributional economic impact of a price increase may be felt by in the domestic market by other stakeholders besides the consumer and producer including; cooperatives, processors, and taxpayers etc. However, this analysis is based on a theoretical framework which assumes that price changes are passed onto only consumers because not all stakeholders are considered in our single sector model.

Our economic welfare model defines supply and demand relationships for a specific commodity, i.e. milk, in a specific time, i.e. over a year, and place, i.e. Scotland, on milk producers and consumers. The scope of our paper is much more modest than looking at the full range of knock-on effects associated with Johne’s. Instead, our model tries to both develop and quantify the intuition behind the immediate impacts in the milk market from a welfare perspective that are not often considered in this context. The transmission of 'spillover effects' to other industries and sectors is needed and an important area of future work. The seminal work of Gardner [64] is a particularly useful framework for examining ‘spillover effects’ in multiple markets simultaneously. The value chain work of Gardner [64] provided theoretical insights into the impact of a policy or shock on stakeholders in a supply chain framework. Other models have been developed capturing multiple and more dynamic impacts, and could be relevant in future explorations of the impact of Johne’s. These models range from single country to global economy models capturing impacts in related sectors [65], and wider economic impacts from disease, through to social accounting matrix (SAM) methods [66]. A multi-market model incorporating demand, as well as supply, and linking related markets to trace the impacts of a change in one market on output, prices, and incomes in related markets in the supply chain is an important area for future research. Our modelling framework could be extended to form the basis of such a multi-market model. Multi-sector approaches such as SAMs could also be employed if such databases are available at sufficiently fine level of disaggregation. These models can generate multipliers [67,68] to quantify how the impact of an exogenous shock, such as a disease outbreak, is likely to be transmitted through an economy [65]. An appropriate level of disaggregation in the is important to avoid exaggeration of the impact of a disease shock. A drawback of SAMs is that they do not allow for price changes that partial equilibrium models are capable of incorporating [65]. Computable general equilibrium (CGE) models optimise how a multi-sector economy might respond to policy changes or a shock, such as an animal disease outbreak, over time until equilibrium is restored but require a greater degree of complexity in terms of development and interpretation of output. The use of CGEs in modelling the intertemporal global impacts of disease associated with international trade [69] is an important recent development in research. For an endemic disease such as Johne’s, which is unlikely to have major impacts on international trade, single country CGE models disaggregated at a regional level within the country may be more appropriate [70,71]. This innovation in CGEs allows for a finer resolution adopting a bottom-up approach capturing a more detailed sector disaggregation and regional breakdown within a country. Such a model could adopted in the future, although appropriate data would be need to be collected and processed for it to be suitable in a Scottish context.

Infected producers also incurred losses suggesting that the higher market price of milk is not sufficient to offset losses incurred from reduced yield and higher production costs. Uninfected producers are the only winners benefitting from maximum attainable output and a higher market price because they do not incur additional production costs associated with the disease. The estimates of net economic surplus with respect to the elasticity of demand and supply at the national-level are relatively narrow for alternative scenarios, suggesting that our estimates are robust given market conditions. Following a supply shock as a result of Johne’s the three stakeholder groups are likely to retain their respective net economic losses/gains. The possibility and severity of a supply shock should be kept in mind when considering alternative disease scenarios and when justifying the management of cattle diseases. From a policy perspective, the magnitude of economic welfare of Johne’s disease can be compared to that of other endemic cattle diseases to act as a decision-support tool to prioritise spending on the compensation to infected producers, and the control and prevention of alternative animal diseases.

The results further suggest that a Johne’s eradication scheme would favour consumers and producers of infected herds who experience an economic welfare loss as a result of Johne’s. However, such a scheme may be unwelcomed among uninfected producers because they might lose their comparative advantage, assuming producers know the correct Johne’s health status of their herd, and depending on who pays the cost of eradication. Johne’s control strategies across endemically affected countries vary in their structure and effectiveness, highlighting the variation in management activities in response to common challenges associated with Johne’s [72]. Denmark and the Netherlands both have a long-term goal to eradicate Johne’s [6,73]. Johne’s disease control measures can increase per capita revenue of dairy farms compared to farms with no Johne’s disease control [74]. However, a barrier cited to eradication is the uncertainty as to the perceived cost-benefit of control activities, which has been cited as one of the main reason affecting farmer participation in eradication [72].

While the analysis in this paper evaluates the distribution of economic welfare associated with a Johne’s disease free herd relative to an infected herd, understanding the cost-effectiveness of different control strategies and who pays for them is a critical area for future research. The economic welfare analysis in this paper presents the economic cost associated with the introduction of Johne’s, the financial cost of containment to prevent MAP from spreading was not considered. However, this cost can be compared to the cost of containment or eradication, but how should such costs be shared along the supply chain? Alternative animal health incentive mechanisms should be considered when considering how the economic cost of eradication is distributed amongst stakeholders. Human and animal health are closely linked and the ultimate benefits to human health support the case for animal health being considered a public good [75] because so a potential benefit of reduced animal disease prevalence is an improvement in public health [76]. Circumstances where a public good element exists can have implications for Government policy in terms of regulation or financial support [77]. If animal health has the characteristics of a public good and the market fails to take into account this element and its associated externalities, as demanded by society, there is a rationale for the public sector to correct this market failure [78]. In the UK, the government sometimes compensate costs associated with livestock culled while infected producers bear the cost of consequential losses. The economic welfare analysis framework presented in this paper provides the basis for policy support, in the case of Johne’s disease, since the estimated economic welfare gains/losses help improve our understanding of distribution of trade-offs between stakeholders and justify investment of taxpayers’ money in animal health.

The benefits of improved control and prevention of Johne’s must be transparent to incentivise farmers to tackle the disease [79]. Otherwise, individual dairy producers are likely to act in self-interest, inclined to let others bear the cost of eradication, leading to the problem of “free-riding”. A rational producer may choose not to participate in a Johne’s disease eradication programme if other livestock producers do, depending on the cost of participating and the anticipated gains or losses associated with the action of others. Therefore, there is a need for collective action, rather than individual-based solutions, in order to improve animal health [78,80]. Studies suggest that farmers are risk-averse [81] in which case dairy farmers might choose to minimise the risk of Johne’s rather than minimise the expected cost when managing the disease. Using economic incentives is important for the collective action in the provision of public goods [82]. Differential pricing incentives are quite common in ensuring food safety. For instance, milk pricing incentives allow uninfected producers to benefit from staying disease-free. In Denmark, milk price differentiation of only €0.005 per litre between accredited and non-accredited herd producers incentivised participation in a milk quality assurance programme [83]. Such a pricing mechanism may be necessary because without it uninfected producers have little incentive to fund eradication. Alternatively, risk financing of livestock diseases based on a levy or insurance system could pool and spread the cost of losses between a larger number of stakeholders [84].

The UK is the third largest producer of milk in the EU [85]. The milk market is an important sector for UK agriculture: in 2014 milk accounted for 17.8% of total agricultural output worth £4.6bn [86]. Only 3% of all UK produced milk is exported, the remainder is produced for the domestic market [85,86]. Approximately 92% of Scottish dairy products are sold in the UK [63]. This over-reliance on the domestic market could leave the UK vulnerable to a supply shock following disease. Coupled with pressures of increasing global population, milk production will need to mirror increasing demand. Scotland is expected to produce 1.6 billion litres of milk a year by 2025, a 50% growth over 10 years which is market-driven by farmers and dairy companies [63]. However, a cow infected with Johne’s is likely to produce less milk [14] which could have trade implications if scaled up to a national-level. International trade and milk quality were not considered in this study. A shortfall in either milk production or quality could lead to a worsening of the trade balance, with respect to milk, because in the worst-case scenario the volume of milk imported might need to increase to compensate for the reduction in domestic production as a result of Johne’s.

**Fig 7:**
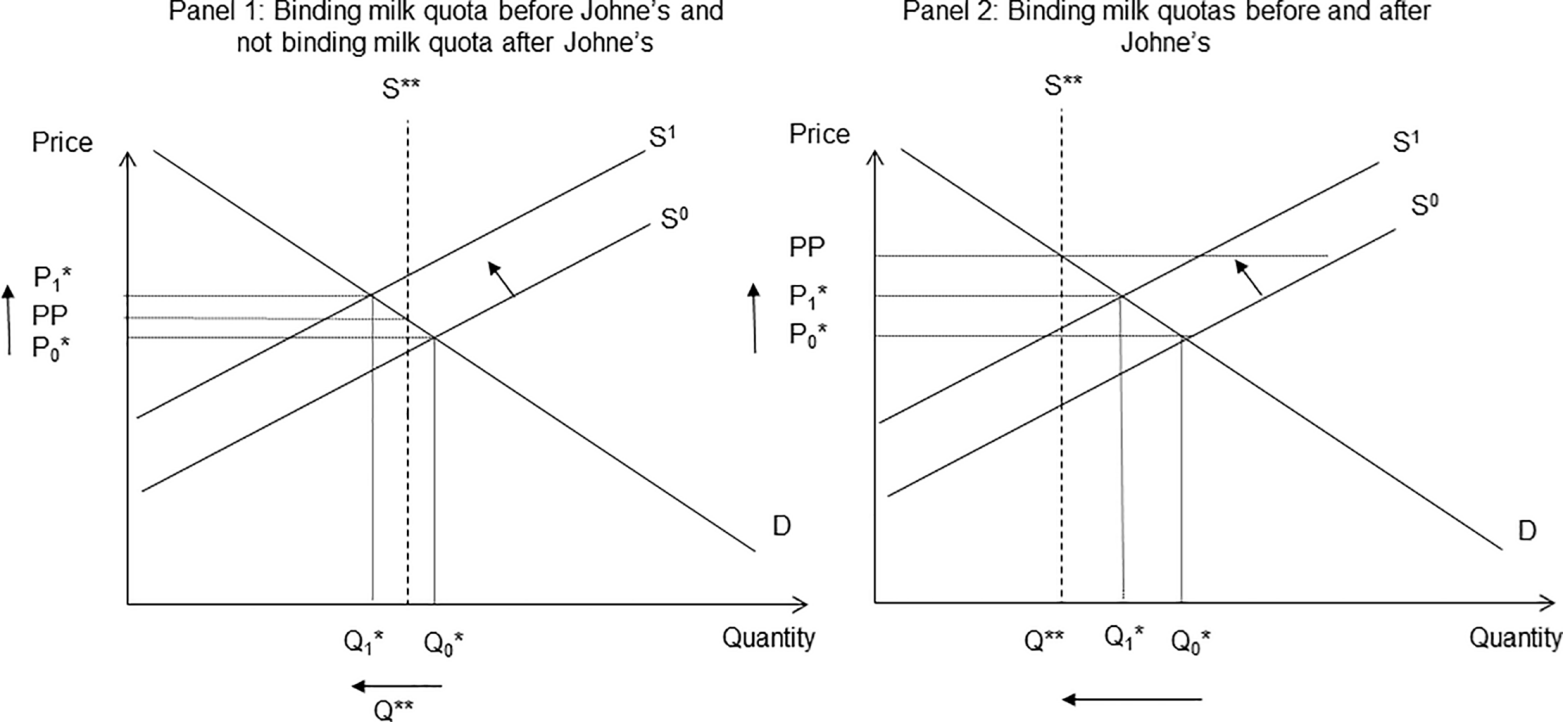
Demand and supply equilibrium associated with binding and non-binding milk quotas. In Fig 7, the intersection of supply curves, S^0^, demand curve, D, and milk quota, S**, determine the initial equilibrium price, P_0_*, and quantity supplied by the market, Q_0_*. A decrease in milk production associated with Johne’s disease shifts the supply curve backward to S^1^. In panel 1 the milk quota is binding before Johne’s because Q_0_*> Q** (quantity associated with milk quota S**), but not binding after Johne’s because Q_1_* < Q**, the price increases from P_0_* to P_1,_* above producer price, PP. In the case of binding milk quotas (panel 2), the equilibrium quantity supplied is restricted to Q** creating a new supply curve (S**), because Q_1_* is above the binding milk quota, and fixed price, PP.

An important assumption in this paper is that milk prices follow “free-market” principles, whereby price is determined by the intersection of supply and demand. In economics a free market does not operate under any restrictions, for example there is no government intervention or regulations. In reality such idealised market assumptions do not necessarily hold in practice and markets are distorted to different degrees. In the EU, agricultural products, including dairy, are subject to a range of different policy measures, including quotas and other types of domestic support that raise and/or artificially fix the producer price. In practice, the UK milk market is distorted by institutional support and does not reflect the true market value of milk [87]. Under the Common Agricultural Policy, milk quotas were introduced in the UK in April 1984 to address oversupply of milk on the EU market. While the impacts of disease will vary somewhat in a distorted market relative to a free market, our assumption of the latter can be justified by a couple of reasons. First, in Scotland, milk quotas were abolished in March 2015 [25], suggesting the milk sector now operates closer to free market conditions [26]. Second, even under the previous quota regime, milk quotas were often not binding. From an economic welfare standpoint, that implies a supply shock as arising from Johne’s would raise prices in the manner assumed by our analysis (see Fig 7, panel 1). If quotas were binding, prices would remain fixed (see Fig 7, panel 2), with the deadweight costs (i.e. loss that is not captured by consumers or producers) of the quota reduced by disease, as less production would be taken offline as a result of policy. This work is not presenting an empirical study, instead it is a first attempt to present a useful framework for estimating the economic welfare of Johne’s disease, which captures the wider economic costs of a disease beyond direct costs at the farm-level. The interactions of public policy (and market power as discussed above) and animal disease remain an under-researched area and would be a worthy area for future research.

Diagnostic tests to detect MAP, at different stages of infection, vary in accuracy [88]. Hence, the national herd prevalence of Johne’s could be reduced with more reliable testing. The incidence of the disease will increase over time if adequate management practices to control Johne’s disease are not implemented [89]. For example, the national herd prevalence of herds affected by Johne’s is likely to have increased since 2006, due to the increased movement of cattle associated with bovine tuberculosis [90]. However, much uncertainty surrounds national herd prevalence due to insufficient and poorly understood data [11]. Up-to-date estimates of animal health parameters, such as prevalence, are often not available in the literature. The most recent estimate for dairy national herd prevalence of Johne’s disease was estimated to be 17.5% ±10% for Great Britain in 2001 [10], with no such estimates for Scotland. However, NMR herd data suggests national herd prevalence to be less than 10%, with the majority of herds believed to have a national herd prevalence of less than 6% [90]. Therefore, much uncertainty surrounds estimates of national herd prevalence. Coupled with this, economic welfare estimates were sensitive to national herd prevalence (Tables 3 and 4). This suggests that uncertainty surrounding the national herd prevalence needs to be minimized in order to draw a more accurate picture of the economic cost of the disease. Expert elicitation can be used to this effect as a systematic approach to synthesize expert opinion in addressing uncertainty associated with limited or unavailable data [91].

Epidemiological data underpin economic welfare models, so expert elicitation of animal health data is a useful tool to inform and support decision-making in relation to the economic cost of alternative animal health scenarios. Expert elicitation can identify knowledge gaps and often represents the only method for synthesising knowledge [92]. Revised estimates of national herd prevalence are important if the economic welfare associated with Johne’s disease is to be estimated with a greater degree of certainty, so that we can better understand and make more informed decisions as to the economic cost of Johne’s. Similarly, a useful extension to this analysis would be examining the longer-term economic welfare impacts of Johne’s based on the divergence in herd demographic structures that would exist in infected versus uninfected herds. As these paths of herd growth could be different, they could compound the losses faced by infected producers and consumers alike, strengthening the case for Johne’s control.

The “tip of the iceberg” effect also poses a problem for the detection of Johne’s and for estimating true national herd prevalence. Due to the nature of Johne’s disease, infected animals shedding MAP are often only a small proportion of the total number of infected animals. In an infected herd it is likely that for every dairy cow exhibiting clinical signs of Johne’s there are 25 animals that are infected but not showing any clinical signs of the disease (i.e. subclinical) [19]. Therefore, the true national herd prevalence is likely to be higher than the observed prevalence, which has a knock-on effect for the economic welfare of producers and consumers alike, who may be experiencing greater losses than expected.

Human and animal health are closely linked, so a potential benefit of reduced animal disease prevalence is an improvement in public health [76]. MAP is similar to Crohn’s disease both clinically and pathologically [93]. There is speculation of a causal link between MAP and Crohn’s disease [94,95] and Johne’s as a potential zoonotic [96]. The thermal resistance of MAP suggests that pasteurisation may not adequately kill the organism [97,98]. However, it is uncertain what danger MAP presents to consumers exposed to dairy or meat products from infected animals [99]. Confirmation of a link between Johne’s and Crohn’s could potentially trigger a reduction in demand whereby infected producers face losses, as would uninfected producers unless a “Johne’s-free” milk certification scheme were introduced. Therefore, the change in the quantity of milk supplied due to Johne’s coupled with a reduction in consumer demand (via a fall in consumer confidence in food safety) remains unclear. Hence, in this study the change in consumer demand following an outbreak of Johne’s was assumed not to change.

## Conclusions

This paper has evaluated the distribution of economic gains and losses amongst stakeholders associated with Johne’s disease. The economic welfare modelling framework presented is a useful tool to support policy decision-making in the evaluation of alternative animal disease scenarios when prioritising public spending on compensation, as well as prevention and control strategies. Economic welfare was sensitive to national herd prevalence, indicating a need for improved testing of Johne’s along with more robust estimates of incidence of the disease on which to estimate the economic impact of Johne’s. The analysis in this paper provides a *ceteris paribus* situation as to the relative distribution of economic welfare following the introduction of Johne’s in Scottish dairy farms, which is a necessary starting point for further evaluation until the national herd prevalence of Johne’s is better understood.

## References

[1] Barkema HW, Hesselink JW, McKenna SL, Benedictus G, Groenendaal H. Global prevalence and economics of infection with *Mycobacteriu*m *avium* subsp *paratuberculosis* in ruminants. In: Behr MA, Collins DM, editors. Paratuberculosis: Organism disease control Wallingford: CAB International; 2010 p. 10–21.

[2] Collins MT. Update on *paratuberculosis*: 1. Epidemiology of Johne's disease and the biology of *Mycobacterium paratuberculosis*. Ir Vet J 2003;56(11):565–574.

[3] Windsor PA, Whittington RJ. Evidence for age susceptibility of cattle to Johne’s disease. Vet J 2010;184(1):37–44. doi: 10.1016/j.tvjl.2009.01.007 19246220 10.1016/j.tvjl.2009.01.007

[4] Larsen AB, Merkal RS, Cutlip RC. Age of cattle as related to resistance to infection with *Mycobacterium paratuberculosis*. Am J Vet Res 1975 3;36(3):255–257. 1115424

[5] Begg DJ, O'brien R, Mackintosh CG, Griffin JF. Experimental infection model for Johne's disease in sheep. Infect Immun 2005;73(9):5603–5611. doi: 10.1128/IAI.73.9.5603-5611.2005 16113277 10.1128/IAI.73.9.5603-5611.2005 PMC1231139

[6] Nielsen SS, Toft N. A review of prevalences of *paratuberculosis* in farmed animals in Europe. Prev Vet Med 2009;88(1):1–14. doi: 10.1016/j.prevetmed.2008.07.003 18817995 10.1016/j.prevetmed.2008.07.003

[7] Carslake D, Grant W, Green LE, Cave J, Greaves J, Keeling M, et al Endemic cattle diseases: comparative epidemiology and governance. Philos Trans R Soc Lond B Biol Sci 2011;366(1573):1975–1986. doi: 10.1098/rstb.2010.0396 21624918 10.1098/rstb.2010.0396 PMC3130389

[8] Vidal-Diez A, Sayers A, Gardner I, Cook A. An integrated strategy to determine the herd level prevalence of Johne’s disease in the UK dairy herd. Final report SB4022 2009:214.

[9] Cetinkaya B, Erdogan H, Morgan K. Prevalence, incidence and geographical distribution of Johne's disease in cattle in England and the Welsh borders. Vet Rec 1998;143:265–265. 9787418 10.1136/vr.143.10.265

[10] Caldow G, Gunn G. Assessment of surveillance and control of Johne’s disease in farm animals in GB. Scottish Agricultural College Veterinary Science Division report 2000:245.

[11] Davidson RS, McKendrick IJ, Wood JC, Marion G, Greig A, Stevenson K, et al Accounting for uncertainty in model-based prevalence estimation: *paratuberculosis* control in dairy herds. BMC Vet Res 2012;8:159 doi: 10.1186/1746-6148-8-159 22963482 10.1186/1746-6148-8-159 PMC3544565

[12] Defra. Guidance on the control of Johne's disease in dairy herds. 2010:1–11.

[13] Pritchard TC, Coffey MP, Bond KS, Hutchings MR, Wall E. Phenotypic effects of subclinical paratuberculosis (Johne’s disease) in dairy cattle. J Dairy Sci 2017;100(1):679–690. doi: 10.3168/jds.2016-11323 27837981 10.3168/jds.2016-11323

[14] Lombard JE, Garry FB, McCluskey BJ, Wagner BA. Risk of removal and effects on milk production associated with *paratuberculosis* status in dairy cows. J Am Vet Med Assoc 2005;227(12):1975–1981. doi: 10.2460/javma.2005.227.1975 16379637 10.2460/javma.2005.227.1975

[15] Ott SL, Wells SJ, Wagner BA. Herd-level economic losses associated with Johne's disease on US dairy operations. Prev Vet Med 1999;40:179–192. 10423773 10.1016/s0167-5877(99)00037-9

[16] Raizman EA, Fetrow J, Wells SJ, Godden SM, Oakes MJ, Vazquez G. The association between *Mycobacterium avium* subsp. *paratuberculosis* fecal shedding or clinical Johne's disease and lactation performance on two Minnesota, USA dairy farms. Prev Vet Med 2007;78(3):179–195. 17118473 10.1016/j.prevetmed.2006.10.006

[17] Bennett R, IJpelaar J. Updated estimates of the costs associated with thirty four endemic livestock diseases in Great Britain: A note. J Agric Econ 2005;56(1):135–144.

[18] Stott AW, Jones GM, Humphry RW, Gunn GJ. Financial incentive to control *paratuberculosis* (Johne's disease) on dairy farms in the United Kingdom. Vet Rec 2005;156(26):825–831. 15980134 10.1136/vr.156.26.825

[19] Whitlock RH, Buergelt C. Preclinical and clinical manifestations of *paratuberculosis* (including pathology). Vet Clin North Am Food Anim Pract 1996;12(2):345–356. 8828109 10.1016/s0749-0720(15)30410-2

[20] FAO. Economic analysis of animal diseases 2016;Food and Agriculture Organization of the United Nations:73.

[21] Mangen M, Burrell A. Who gains, who loses? Welfare effects of classical swine fever epidemics in the Netherlands. Eur Rev Agric Econ 2003;30(2):125–154.

[22] Pendell DL, Leatherman J, Schroeder TC, Alward GS. The economic impacts of a foot-and-mouth disease outbreak: a regional analysis. Journal of agricultural and applied economics 2007;39(s1):19–33.

[23] Bennett R. The ‘direct costs’ of livestock disease: the development of a system of models for the analysis of 30 endemic livestock diseases in Great Britain. J Agric Econ 2003;54(1):55–71.

[24] Weldegebriel HT, Gunn GJ, Stott AW. Evaluation of producer and consumer benefits resulting from eradication of bovine viral diarrhoea (BVD) in Scotland, United Kingdom. Prev Vet Med 2009;88(1):49–56. doi: 10.1016/j.prevetmed.2008.07.001 18937987 10.1016/j.prevetmed.2008.07.001

[25] BBC. Milk: End of EU quota heightens UK farmers' fears. BBC news website 2015.

[26] Costa‐Font M, Revoredo‐Giha C. An empirical analysis of UK milk contract prices 2004–2016. Agribusiness 2018.

[27] Pritchett J, Thilmany D, Johnson K. Animal disease economic impacts: A survey of literature and typology of research approaches. International Food and Agribusiness management review 2005;8(1):23–45.

[28] Hueth D, Just R, Schmitz A. Applied welfare economics and public policy Englewood Cliffs, NJ: Prentice-Hall; 1982.

[29] Bannock G, Baxter R, Davis E. The pengiun dictionary of economics 5th ed London: Penguin; 1992.

[30] Bellman R. Dynamic Programming (DP) Princeton: Princeton University Press; 1957.

[31] AHDB. Average UK milk yield. 2015; Available at: www.dairyco.org.uk/market-information/farming-data/milk-yield/average-milk-yield/. Accessed November, 2015.

[32] Benedictus G, Dijkhuizen AA, Stelwagen J. Economic losses due to *paratuberculosis* in dairy cattle. Vet Rec 1987;121(7):142–146. 3660545 10.1136/vr.121.7.142

[33] Agrawal R, Heady E. Markov chain processes In: Agrawal R, Heady E, editors. Methods for Agricultural Decisions Ames, IA: Iowa State University Press; 1972 p. 179–194.

[34] Wilson DJ, Rossiter C, Han H, Sears P. Financial effects of *Mycobacterium paratuberculosis* on mastitis, milk production, and cull rate in clinically normal cows. Agri-Practice 1995;16:12–18.

[35] Wood P. Algebraic model of the lactation curve in cattle. Nature 1967;216(5111):164–165.

[36] SAC. The farm management handbook 2012/13 33rd ed Midlothian: Scotland's Rural College; 2012.

[37] Marcé C, Beaudeau F, Bareille N, Seegers H, Fourichon C. Higher non-return rate associated with *Mycobacterium avium* subspecies *paratuberculosis* infection at early stage in Holstein dairy cows. Theriogenology 2009;71(5):807–816. doi: 10.1016/j.theriogenology.2008.10.017 19117602 10.1016/j.theriogenology.2008.10.017

[38] SAC. The farm management handbook 2014/15 35th ed Midlothian: Scotland's Rural College; 2014.

[39] Forbes D, Gayton S, McKeogh B. Improving the longevity of cows in the UK dairy herd. Milk Development Council 1999.

[40] Van Arendonk J. A model to estimate the performance, revenues and costs of dairy cows under different production and price situations. Agric Syst 1985;16(3):157–189.

[41] Groenendaal H, Nielen M, Jalvingh AW, Horst SH, Galligan DT, Hesselink JW. A simulation of Johne’s disease control. Prev Vet Med 2002;54:225–245. 12114011 10.1016/s0167-5877(02)00027-2

[42] R Core Team. R: A language and environment for statistical computing. 2015.

[43] Oanda. Average exchange rates. 2017; Available at: www.oanda.com/currency/average. Accessed February, 2017.

[44] Revoredo-Giha C. SRUC-FMR Food supply model for Scotland. 2017;Economic report produced as part of the 2016–21 Scottish Government Strategic Research Programme. Theme 3 (Food and Health), WP 3.3.1 (Food security, consumption and trade).

[45] Revoredo-Giha C. Demand elasticities by UK country. 2017;Economic report produced as part of the 2016–21 Scottish Government Strategic Research Programme. Theme 3 (Food and Health), WP 3.3.1 (Food security, consumption and trade).

[46] National Records of Scotland. Estimates of households and dwellings in Scotland, 2014 A National Statistics publication for Scotland 2015:1–55.

[47] ONS. Annual mid-year population estimates, 2014 Statistical Bulletin 2015:1–15.

[48] Scottish Government. Results from the June 2014 Scottish Agricultural Census A National Statistics Publication for Scotland 2014:1–52.

[49] Lichtenberg E, Parker DD, Zilberman D. Marginal analysis of welfare costs of environmental policies: the case of pesticide regulation. Am J Agric Econ 1988;70(4):867–874.

[50] Stott A, Milne C, Peddie S, Gunn G. Balancing public and private interests in EU food production. 56th Annual Meeting of European Association of Animal Production 2005 June 5–8.

[51] ParaTBTools. Development of improved tools for detection of paratuberculosis in livestock, M. paratuberculosis in livestock, M. paratuberculosis in food and for the risk of human exposure. 2017; Available at: https://www.visavet.es/paratbtools/index.php. Accessed January, 2017.

[52] Losinger WC. Economic impacts of reduced milk production associated with epidemiological risk factors for Johne's disease on dairy operations in the USA. J Dairy Res 2006;73(1):33–43. doi: 10.1017/S0022029905001378 16433959 10.1017/S0022029905001378

[53] Lovell R, Levi M, Francis J. Studies on the survival of Johne's bacilli. J Comp Pathol 1944;54:120–129.

[54] Havrila A, Arch A. Pesticide regulations: Measuring welfare costs with marginal analysis. 35th Annual Conference Australian Agricultural Economics Society (AARES) 1991 February 11–14:24.

[55] Nicholson W, Snyder C. Microeconomic theory: Basic principles and extensions 6th ed Forth Worth TX, USA: Dryden Press; 1995.

[56] Andersson H, Lexmon Å, Robertsson J, Lundeheim N, Wierup M. Agricultural policy and social returns to eradication programs: the case of Aujeszky's disease in Sweden. Prev Vet Med 1997;29(4):311–328. 9234439 10.1016/s0167-5877(96)01073-2

[57] Ebel ED, Hornbaker RH, Nelson CH. Welfare effects of the national *pseudorabies* eradication program. Am J Agric Econ 1992;74(3):638–645.

[58] Forsythe KW, Corso B. Welfare effects of the national *pseudorabies* eradication program: Comment. Am J Agric Econ 1994;76(4):968–971.

[59] Colman D, Harvey D. The future of UK dairy farming. Report commissioned jointly by the MDC, DIAL and Defra 2004:28.

[60] Thompson D, Aldritt R, Bolton-Smith C, Buttriss J, Chesher A, Church S, et al. National food survey 2000. Report for Defra by the National Food Survey Committee 2002:213.

[61] BBC. Tesco to fight dairy price-fixing fine from OFT. BBC news website 2011.

[62] ONS. RPI: Ave price—Milk: Pasteurised, per pint. 2018; Available at: https://www.ons.gov.uk/economy/inflationandpriceindices/timeseries/cznt/mm23.

[63] Withers J. Scottish dairy review: “Ambition 2025” report & recommendations. Report to the Scottish Government 2013:15.

[64] Gardner BL. The farm-retail price spread in a competitive food industry. Am J Agric Econ 1975;57(3):399–409.

[65] Rich K, Miller G, Winter-Nelson A. A review of economic tools for the assessment of animal disease outbreaks. Revue Scientifique Et Technique-Office International Des Epizooties 2005;24(3):833. 16642754

[66] Rich KM, Roland-Holst D, Otte J. An assessment of the ex-post socio-economic impacts of global rinderpest eradication: Methodological issues and applications to rinderpest control programs in Chad and India. Food Policy 2014;44:248–261.

[67] Rich KM, Winter-Nelson A, Miller GY. Enhancing economic models for the analysis of animal disease. Rev Sci Tech 2005 12;24(3):847–856. 16642755

[68] Miller RE, Blair PD. Input-output analysis: foundations and extensions: Cambridge University Press; 2009.

[69] Van Ha P, Kompas T, Nguyen HTM, Long CH. Building a better trade model to determine local effects: A regional and intertemporal GTAP model. Econ Model 2017;67:102–113.

[70] Adams P, Horridge M, Wittwer G. MMRF-Green: a dynamic multi-region applied general equilibrium model of the Australian economy, based on the MMR and MONASH models Centre for Policy Studies, Monash University 2002.

[71] Van Ha P, Nguyen HTM, Kompas T, Che TN, Trinh B. Rice production, trade and the poor: regional effects of rice export policy on households in Vietnam. Journal of Agricultural Economics 2015;66(2):280–307.

[72] Geraghty T, Graham DA, Mullowney P, More SJ. A review of bovine Johne's disease control activities in 6 endemically infected countries. Prev Vet Med 2014;116(1):1–11. 24997766 10.1016/j.prevetmed.2014.06.003

[73] Benedictus G, Verhoeff J, Schukken Y, Hesselink J. Dutch *paratuberculosis* programme history, principles and development. Vet Microbiol 2000;77(3):399–413. 11118725 10.1016/s0378-1135(00)00325-4

[74] Massaro T, Lenhart S, Spence M, Drakes C, Yang G, Agusto F, et al Modeling for cost analysis of Johne's disease control based on EVELISA testing. J Biol Syst 2013;21(4):1340010.

[75] WHO-OIE. Operational framework for good governance at the human-animal interface: Bridging WHO and OIE tools for the assessment of national capacities. World Health Organisation, World Organisation for Animal Health, and World Bank Group report 2014:86.

[76] Badiola J, Bakker D, Garcia Marin J, Gilot P, Hermon-Taylor J, Sharp J, et al. Possible links between Crohn's disease and Paratuberculosis. Report of the Scientific Committee on Animal Health and Animal Welfare Adopted 2000:76.

[77] FAWC. Economics and farm animal welfare. Farm Animal Welfare Committee report 2011:49.

[78] Bennett R. Economic rationale for interventions to control livestock disease. EuroChoices 2012;11(2):5–11.

[79] NML. Johne’s disease testing toolkit: frequently asked questions. National Milk Laboratories report 2011:12.

[80] Rushton J, Leonard D. The new institutional economics and the assessment of animal disease control In: Rushton J, editor. The economics of animal health and production Wallingford, UK: CABI International; 2009 p. 144–148.

[81] Oglethorpe DR. Sensitivity of farm plans under risk-averse behaviour: A note on the environmental implications. J Agric Econ 1995;46(2):227–232.

[82] Olson M. The logic of collective action: Public goods and the theory of groups 1st ed Cambridge: Harvard University Press; 1965.

[83] Velthuis A, Weber M, de Koeijer A, van Roermund H. Milk-quality-assurance program for Johne's disease: Decision analysis from a farmers' perspective. Proceedings of the 11th International Symposium on Veterinary Epidemiology and Economics (ISVEE) 2006 8 7–11:313.

[84] van Asseldonk M, Meuwissen M, Huirne R. A risk financing model for livestock epidemics in the European Union Institute for Risk Management in Agriculture (IRMA), Wageningen University and Health and Consumer Protection DG, European Commission 2003:55.

[85] Bate A. UK dairy industry statistics. Briefing paper no. 2721 2016:10.

[86] National Statistics. Agriculture in the United Kingdom 2014. Report produced by Department for Environment, Food & Rural Affairs, Department of Agriculture & Rural Development (Northern Ireland), Welsh Assembly, The Department for Rural Affairs & Heritage, & the Scottish Government, Rural & Environment Research 2015:99.

[87] Bennett R, Christiansen K, Clifton-Hadley R. Preliminary estimates of the direct costs associated with endemic diseases of livestock in Great Britain. Prev Vet Med 1999;39(3):155–171. 10327436 10.1016/s0167-5877(99)00003-3

[88] Britton LE, Cassidy JP, O'Donovan J, Gordon SV, Markey B. Potential application of emerging diagnostic techniques to the diagnosis of bovine Johne's disease (*paratuberculosis*). Vet J 2016;209:32–39. doi: 10.1016/j.tvjl.2015.10.033 26831164 10.1016/j.tvjl.2015.10.033

[89] Stabel JR. On-farm batch pasteurization destroys *Mycobacterium paratuberculosis* in waste milk. J Dairy Sci 2001;84(2):524–527. doi: 10.3168/jds.S0022-0302(01)74503-1 11233038 10.3168/jds.S0022-0302(01)74503-1

[90] NMR. A veterinary guide to Johne's testing strategies on the dairy farm. Report by the National Milk Records 2012:26.

[91] Runge MC, Converse SJ, Lyons JE. Which uncertainty? Using expert elicitation and expected value of information to design an adaptive program. Biol Conserv 2011;144(4):1214–1223.

[92] Butler AJ, Thomas MK, Pintar KD. Systematic review of expert elicitation methods as a tool for source attribution of enteric illness. Foodborne Pathog Dis 2015;12(5):367–382. doi: 10.1089/fpd.2014.1844 25826450 10.1089/fpd.2014.1844

[93] McKenna SL, Keefe GP, Tiwari A, VanLeeuwen J, Barkema HW. Johne's disease in Canada part II: Disease impacts, risk factors, and control programs for dairy producers. Can Vet J 2006;47(11):1089 17147140 PMC1624920

[94] Uzoigwe JC, Khaitsa ML, Gibbs P. Epidemiological evidence for *Mycobacterium avium* subspecies *paratuberculosis* as a cause of Crohn's disease. Epidemiol Infect 2007;135(7):1057–1068. doi: 10.1017/S0950268807008448 17445316 10.1017/S0950268807008448 PMC2870686

[95] Gitlin L, Borody TJ, Chamberlin W, Campbell J. *Mycobacterium avium* ss *paratuberculosis*-associated diseases: piecing the Crohn's puzzle together. J Clin Gastroenterol 2012;46(8):649–655. doi: 10.1097/MCG.0b013e31825f2bce 22858515 10.1097/MCG.0b013e31825f2bce

[96] Atreya R, Bülte M, Gerlach G, Goethe R, Hornef MW, Köhler H, et al Facts, myths and hypotheses on the zoonotic nature of *Mycobacterium avium* subspecies *paratuberculosis*. Int J Med Microbiol 2014;304(7):858–867. doi: 10.1016/j.ijmm.2014.07.006 25128370 10.1016/j.ijmm.2014.07.006

[97] Rowe M, Grant I, Dundee L, Ball H. Heat resistance of *Mycobacterium avium* subsp. *paratuberculosis* in milk. Irish J Agr Food Res 2000;39(2):203–208.

[98] Grant IR, Rowe MT, Dundee L, Hitchings E. *Mycobacterium avium* ssp. *paratuberculosis*: its incidence, heat resistance and detection in milk and dairy products. Int J Dairy Technol 2001;54(1):2–13.

[99] Stabel J. Johne's disease and milk: do consumers need to worry? J Dairy Sci 2000;83(7):1659–1663. doi: 10.3168/jds.S0022-0302(00)75034-X 10908069 10.3168/jds.S0022-0302(00)75034-X

