# Correction: A framework for estimating society's economic welfare following the introduction of an animal disease: The case of Johne's disease

**DOI:** 10.1371/journal.pone.0202253

**Published:** 2018-08-09

**Authors:** Alyson S. Barratt, Matthieu H. Arnoult, Bouda Vosough Ahmadi, Karl M. Rich, George J. Gunn, Alistair W. Stott

Figs 2–6 are missing figure legends. Additionally, the captions for Figs 2–6 are incomplete. Please see updated figs and captions here.

**Fig 2:**
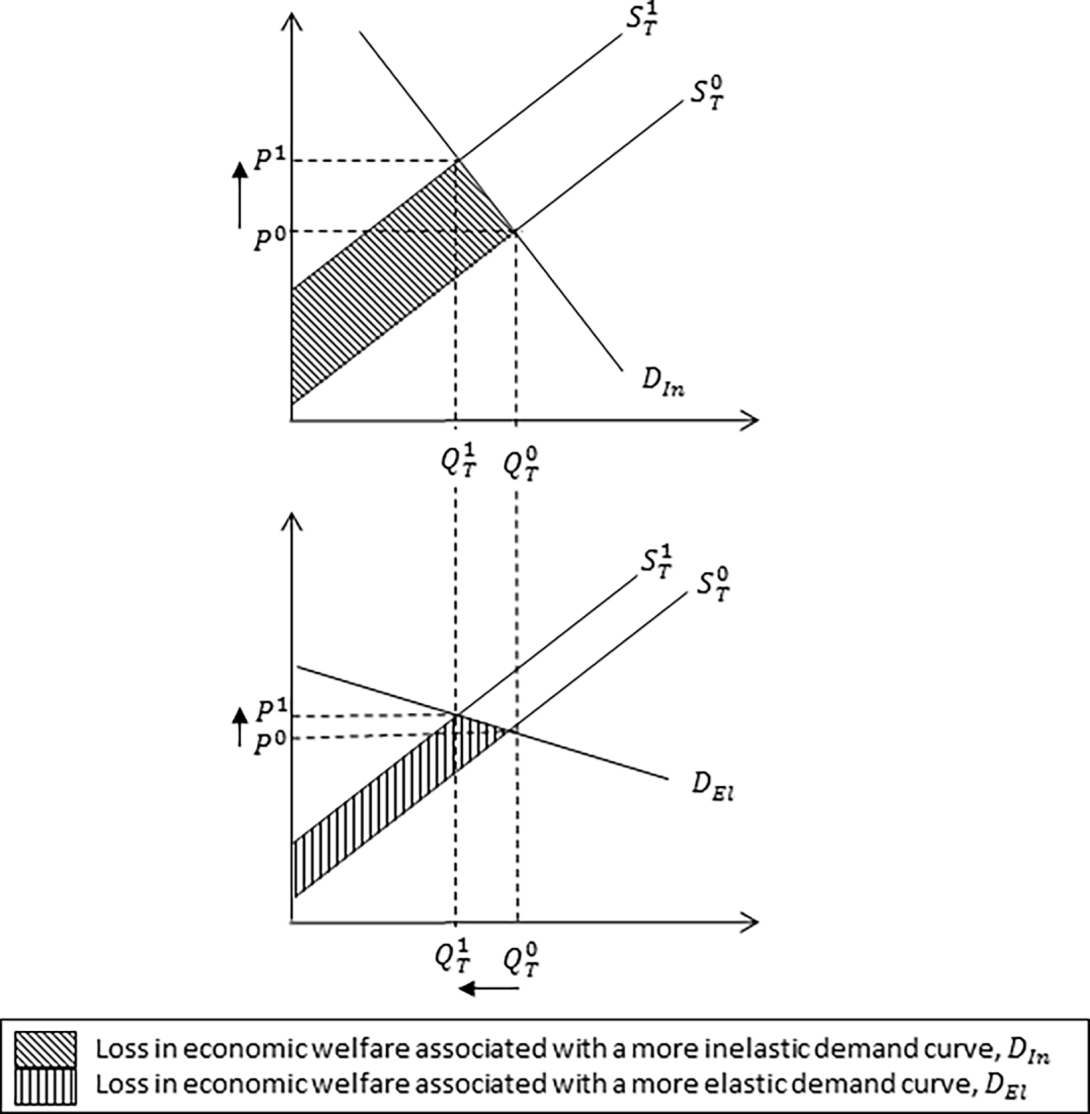
Impact of an inelastic and elastic demand curve on equilibrium market price and quantity. The impact of an inelastic and elastic demand curve on equilibrium market price and quantity associated with a reduction in milk production following an outbreak of Johne’s disease. The inelastic, *D*_*In*_, and elastic, *D*_*El*_, demand curve determine the responsiveness of consumers to new equilibrium market price, *P*^*1*^. A more inelastic demand curve, *D*_*In*_, (i.e. the demand curve is steeper in shape) reflects a larger loss in economic welfare relative to a relatively more elastic demand curve, *D*_*El*_.

**Fig 3:**
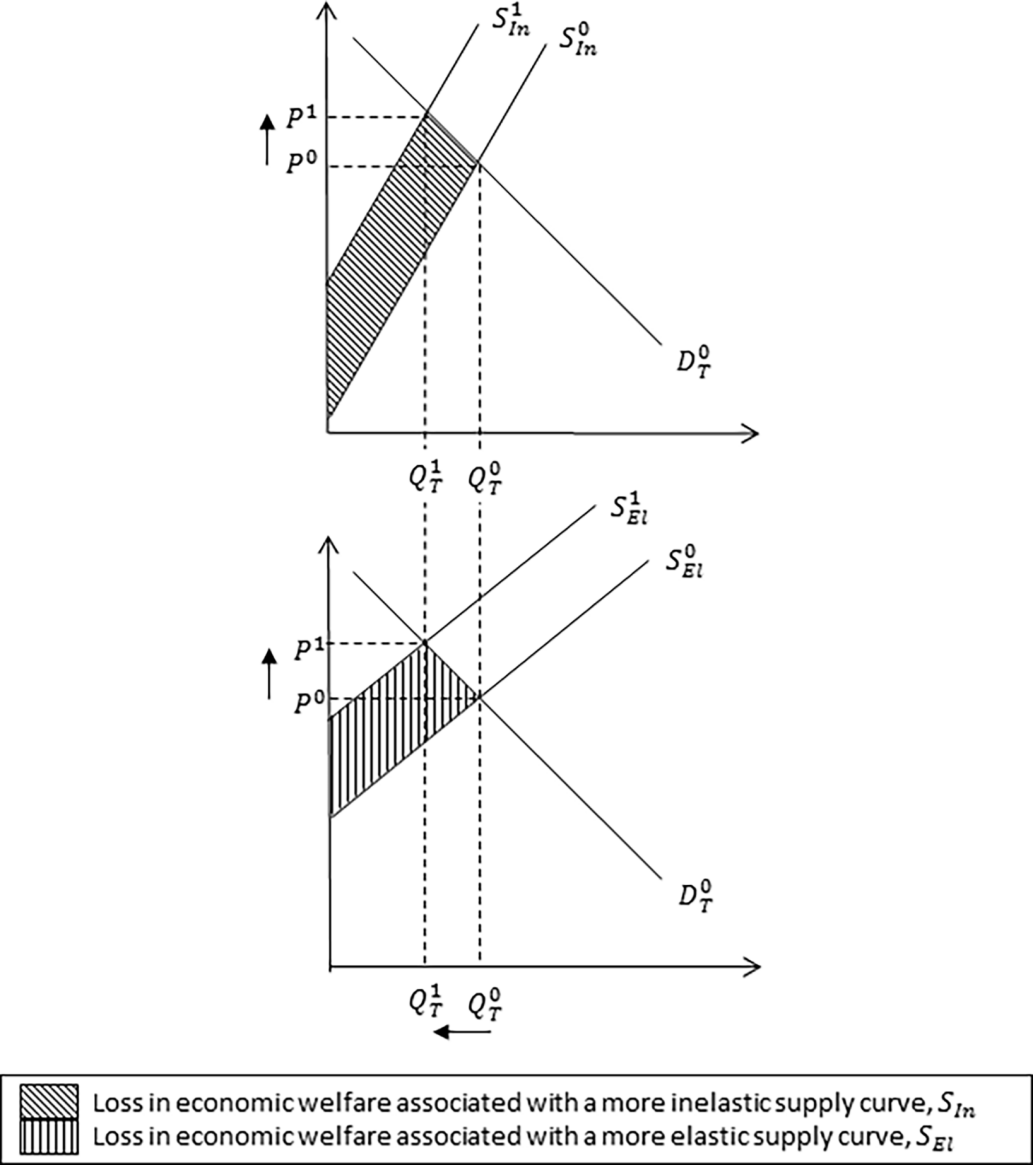
Impact of an inelastic and elastic supply curve on equilibrium market price and quantity. The impact of an inelastic and elastic supply curve on equilibrium market price and quantity associated with a reduction in milk production following an outbreak of Johne’s disease. The inelastic, *S*_*In*_, and elastic, *S*_*El*_, supply curves determine the responsiveness of producers to new equilibrium market price, *P*^1^. A more inelastic supply curve, *S*_*In*_, (i.e. the supply curve is steeper in shape) reflects a larger loss in economic welfare relative to a relative more elastic supply, *S*_*El*_.

**Fig 4:**
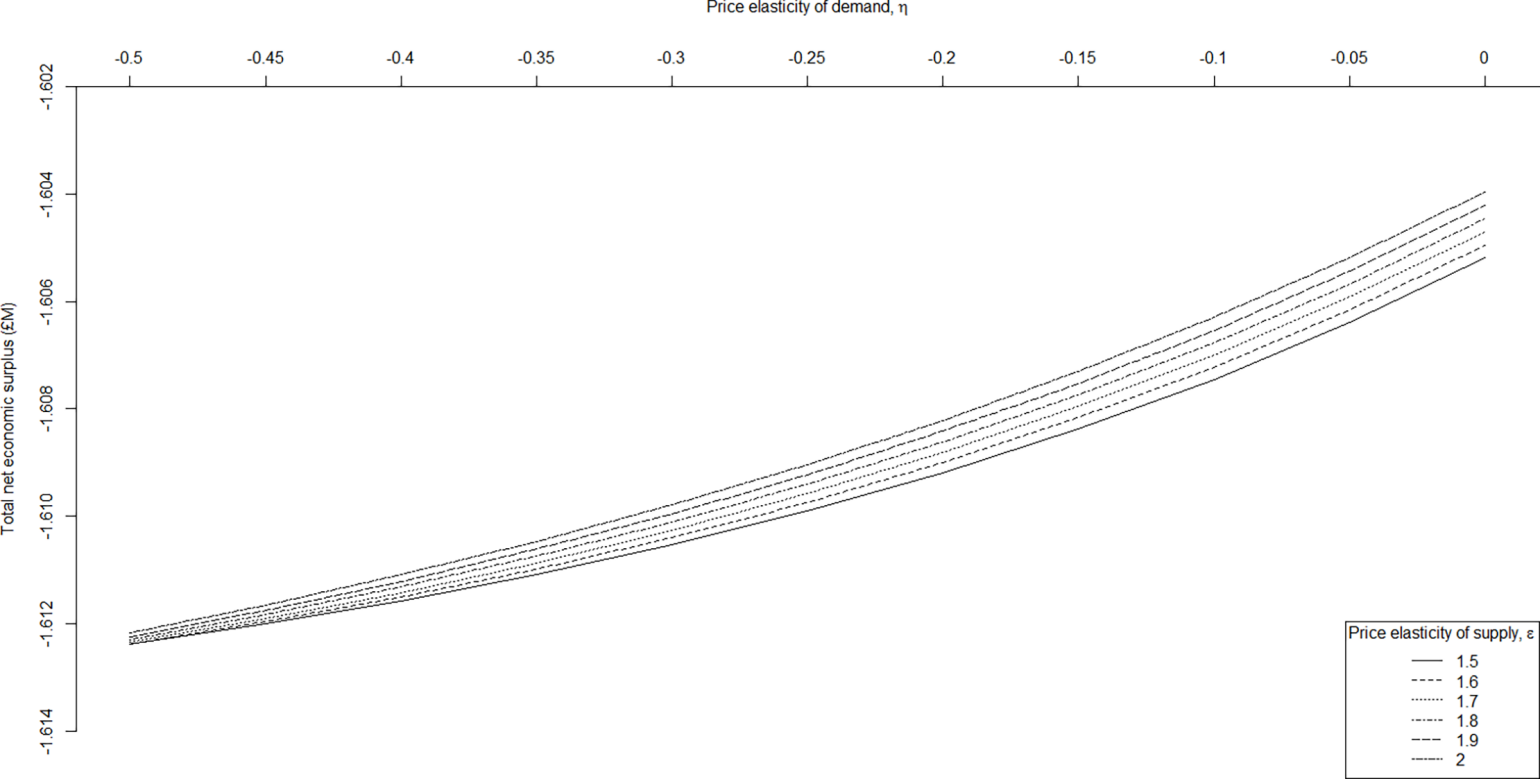
Sensitivity of net economic surplus for Scotland to price elasticity of demand and supply. The sensitivity of aggregated net economic surplus (million £) for Scotland following an outbreak of Johne’s with respect to variation in the price elasticity of demand, *η*, (-0.50 to 0.00), and price elasticity of supply, *ε*, (i.e. 1.5, 1.6, 1.7, 1.8, 1.9, 2.0).

**Fig 5:**
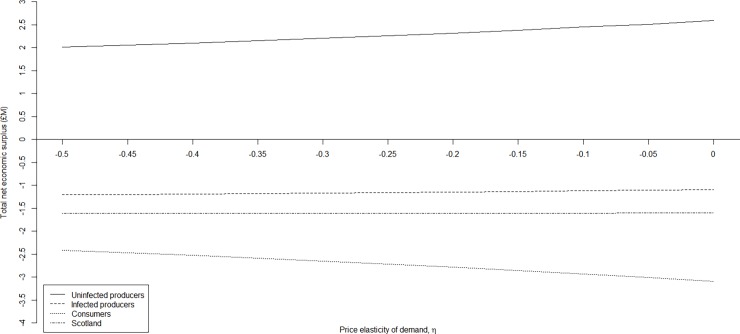
Sensitivity of net economic surplus to the price elasticity of demand by stakeholder group. The sensitivity of net economic surplus (million £) to price elasticity of demand by stakeholder group (i.e. uninfected producers, infected producers, consumers, and Scotland) following an outbreak of Johne’s with respect to a constant price elasticity of supply, *ε*, (1.759), and a variation in the price elasticity of demand, *η*, (i.e. -0.45 to 0.00).

**Fig 6:**
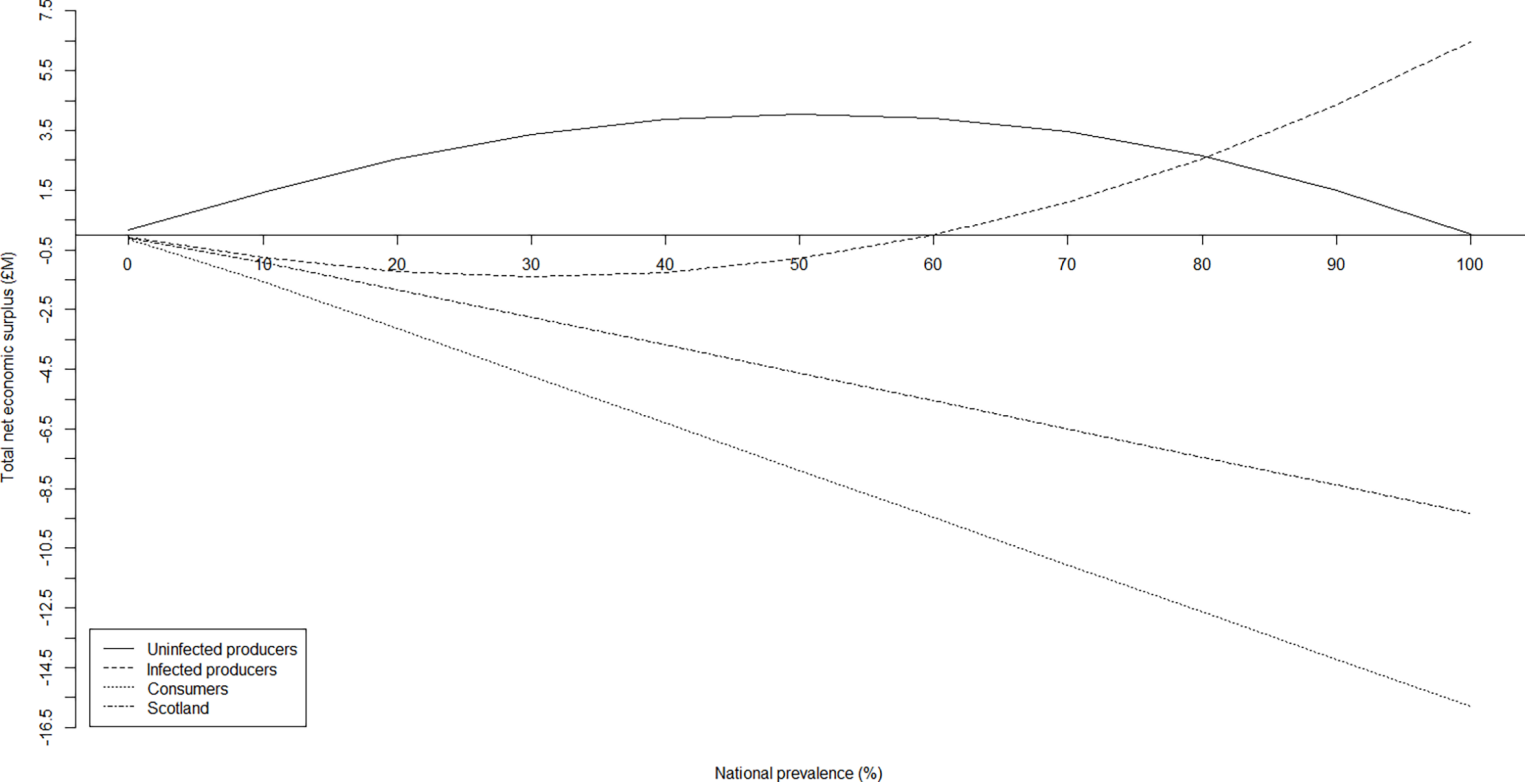
Sensitivity of net economic surplus to national herd prevalence by stakeholder group. The sensitivity of net economic surplus (million £) to national herd prevalence by stakeholder group (i.e. uninfected producers, infected producers, consumers, and Scotland) following an outbreak of Johne’s with respect to a constant price elasticity of demand, *η*, (-0.2198), and price elasticity of supply, *ε*, (1.759).
